# Expanding the spectrum of novel candidate genes using trio exome sequencing and identification of monogenic cause in 27.5% of 320 families with steroid-resistant nephrotic syndrome

**DOI:** 10.1016/j.gendis.2024.101280

**Published:** 2024-03-28

**Authors:** Ronen Schneider, Shirlee Shril, Florian Buerger, Konstantin Deutsch, Kirollos Yousef, Camille N. Frank, Ana C. Onuchic-Whitford, Thomas M. Kitzler, Youying Mao, Verena Klämbt, Muhammad Y. Zahoor, Katharina Lemberg, Amar J. Majmundar, Bshara Mansour, Ken Saida, Steve Seltzsam, Caroline M. Kolvenbach, Lea Maria Merz, Nils D. Mertens, Tobias Hermle, Nina Mann, Dalia Pantel, Abdul A. Halawi, Aaron Bao, Luca Schierbaum, Sophia Schneider, Daanya Salmanullah, Iddo Z. Ben-Dov, Itamar Sagiv, Loai A. Eid, Hazem Subhi H. Awad, Muna Al Saffar, Neveen A. Soliman, Marwa M. Nabhan, Jameela A. Kari, Sherif El Desoky, Mohamed A. Shalaby, Said Ooda, Hanan M. Fathy, Shrikant Mane, Richard P. Lifton, Michael J.G. Somers, Friedhelm Hildebrandt

**Affiliations:** a Department of Pediatrics, Boston Children's Hospital, Harvard Medical School, Boston, MA 02115, USA; b Division of Renal Medicine, Department of Medicine, Brigham and Women's Hospital, Harvard Medical School, Boston, MA 02115, USA; c Institute of Biochemistry & Biotechnology, University of Veterinary & Animal Sciences, Lahore 54000, Pakistan; d Institute of Anatomy, Medical Faculty, University of Bonn, Bonn D-53113, Germany; e Department of Pediatrics, University Hospital Leipzig, Leipzig 04103, Germany; f Institute of Human Genetics, Heidelberg University, Heidelberg 69117, Germany; g Department of Nephrology and Hypertension, Hadassah Medical Center and the Faculty of Medicine, Hebrew University of Jerusalem, Jerusalem 91120, Israel; h Pediatric Nephrology Department, Dubai Hospital, Dubai 14660, United Arab Emirates; i Department of Pediatrics, United Arab Emirates University, Abu Dhabi 15551, United Arab Emirates; j Department of Pediatrics, Center of Pediatric Nephrology & Transplantation, Kasr Al Ainy School of Medicine, Cairo University, Cairo 11562, Egypt; k Egyptian Group for Orphan Renal Diseases (EGORD), Cairo 11451, Egypt; m Department of Pediatrics, Faculty of Medicine, King Abdulaziz University, Jeddah 21589, Saudi Arabia; n Pediatric Nephrology Center of Excellence, King Abdulaziz University Hospital, Jeddah 21589, Saudi Arabia; o Experimental and Clinical Internal Medicine Department, Medical Research Institute, Alexandria University, Alexandria 21511, Egypt; p Department of Pediatrics, Faculty of Medicine, Alexandria University, Alexandria 21526, Egypt; q Department of Genetics, Yale University School of Medicine, New Haven, CT 06510, USA

Steroid-resistant nephrotic syndrome (SRNS) is a leading cause of pediatric end-stage renal disease. Monogenic causes have been detected in 11%–45% of pediatric SRNS using exome sequencing,^1^, ^2^, ^3^ leaving a large proportion of cases without a molecular diagnosis. Here, we report employing trio exome sequencing analysis to detect established and novel causes of SRNS in an international cohort of 320 unrelated families with pediatric SRNS. In 88/320 families (27.5%), exome sequencing revealed a pathogenic variant in a known monogenic SRNS gene. In 18 families (5.6%), we detected a causative variant in a phenocopy gene. In families without a molecular diagnosis, exome sequencing data was evaluated for variants in novel genes. In 18.1% (58/320) of families, we detected variants in a single novel candidate gene for SRNS. In non-consanguineous families, trio analysis increased the rate of novel candidate gene discovery from 15.9% in singlets to 28.8% in duos/trios by detection of deleterious compound heterozygous and *de novo* rare variants (Fig. 1).

**Figure 1:**
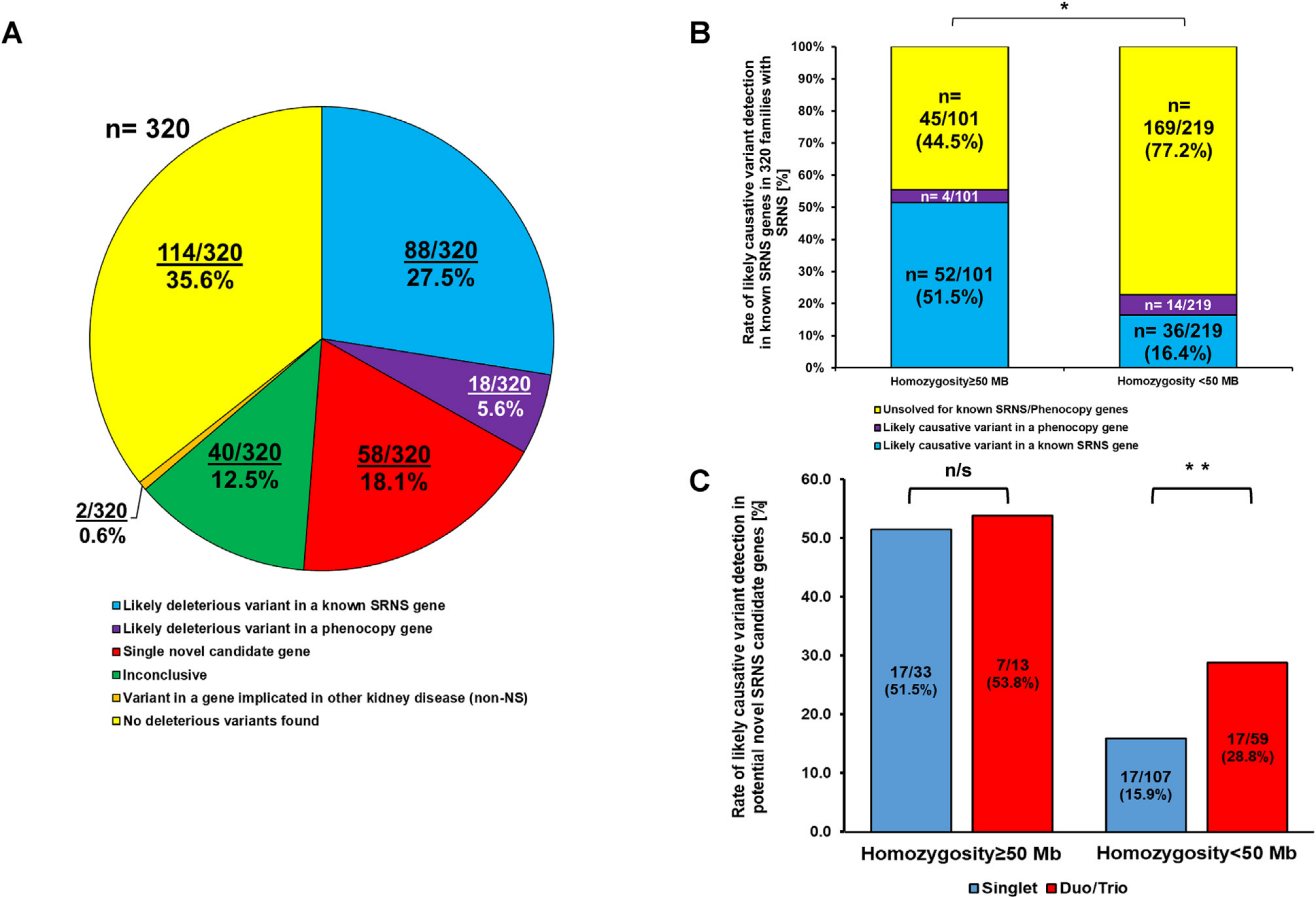
Exome sequencing analysis results in 320 families with SRNS. **(A)** In 88 of 320 (27.5%) families with SRNS, a likely causative variant was detected in one of 27 genes known to cause SRNS if mutated (blue). In 5.6% of families, a likely causative variant was found in genes causing a kidney disease that may represent phenocopies of SRNS (purple). In 18.1% of families, a variant in one potential novel candidate gene was identified (red), and in 12.5% variants in more than one potential candidate gene were identified, and hence the genetic analysis was inconclusive (green). In 35.6% of families, no causative variants or candidate genes were detected (yellow). In 0.6% of families, a causative gene for a non-nephrotic syndrome kidney disease was detected. **(B)** The detection rate of likely causative genetic variants in known SRNS genes is dependent on the extent of homozygosity by descent. We examined each family's whole exome sequencing data for the degree of homozygosity by descent (Hildebrandt PLoS Genet 5:p.e1000353, 2019) and defined homozygosity by descent (HBD) to an individual if ≥ 50 Mb of homozygosity were found by mapping. We identified 101/320 (31.5%) families with HBD, in 52 (51.5%) of whom we detected a likely causative variant in an SRNS disease-causing gene. In contrast, in families with <50 Mbp of homozygosity on mapping, a likely causative variant was detected in an SRNS gene in only 36/219 (16.4%). The difference in variant detection between homozygous and non-homozygous families was statistically significant using Fisher's exact test (∗*P* < 0.001). **(C)** Potential novel candidate SRNS genes can be detected at a higher rate using “trio-analysis” than “singlet-analysis” in non-homozygous families. In 45 families with homozygosity of ≥50 Mb by mapping for whom no variants were found in either known SRNS or phenocopy genes, we detected novel candidate SRNS genes in 17 out of 33 ″singlets” (51.5%), and in 7/13 (53.8%) by trio/duo analysis (*P* > 0.05, Fisher Exact Test). In 166 families with homozygosity by mapping of <50 Mb, for whom no variants were found in either known SRNS or phenocopy genes, we detected novel candidate SRNS genes in 17 out of 107 ″singlets” (15.9%), and in 17/59 (28.8%) by trio/duo analysis (∗∗*P* < 0.05, Fisher Exact Test). Of these, 9 were compound-heterozygously inherited, 2 were heterozygous *de novo*, and 6 were homozygously inherited variants. SRNS, steroid-resistant nephrotic syndrome.

To date, more than 59 different genes have been associated with Mendelian genetic SRNS.^4^^,^^5^ A causative genetic variant has been detected in 11%–45% of patients with SRNS/FSGS with onset before age 25 years.^1^, ^2^, ^3^ The variation in detection rate was associated with age of disease onset and subject consanguinity.^1^, ^2^, ^3^ For example, our previous studies employing targeted panel or exome sequencing identified a genetic cause in 29.5% and 25%, respectively, in ∼2000 SRNS families, of which 25% were consanguineous.^1^^,^^2^ In contrast, we detected pathogenic variants in a causative SRNS gene in only 11.1% of pediatric SRNS families consisting of local outbred subjects (9.7% of families were consanguineous).^3^ In the current study, 320 unrelated and ethnically diverse families (343 individuals) with pediatric-onset SRNS before age 25 years were recruited from 1998 to 2018 (Table S1, 2). We performed exome sequencing from DNA samples from these families, and initially evaluated the exome sequencing data for variants in 59 genes associated with pediatric SRNS (Table S3). Complete cohort characteristics and variant filtering approach can be found in the supplementary materials (Tables S1, 2, 9, 10). By this approach, we identified a pathogenic or likely pathogenic variant in an SRNS-associated gene in 27.5% of this cohort (Fig. 1A and Table S6), consistent with our previous work.^1^^,^^2^ Discovery rate was negatively correlated with the age of SRNS onset (Fig. S1) while positively correlated with the degree of parental consanguinity (Fig. 1B). Additionally, we evaluated variants in seven phenocopy genes, associated with traits overlapping with SRNS (*e.g.*, nephrotic range proteinuria, renal dysfunction, end-stage kidney disease). By this approach, we identified a deleterious phenocopy gene variant in 18 families (5.6%) (Fig. 1A and Table S5).

However, no likely pathogenic/pathogenic variants were identified in 212/320 (67%) families. Therefore, we applied multiple genetic hypotheses to identify candidate disease variants in the 212 families lacking a molecular diagnosis.

We hypothesized that trio exome sequencing analysis would increase the detection of candidate genetic variants. We only considered variants that met our genetic filtering criteria (Table S4). Using the variant calling filtering process described in the supplementary material, we employed two approaches to detect variants in potential novel candidate genes for SRNS. i) Singlet analysis: If parental DNA was not available (*n* = 207), we analyzed the proband and looked only for variants inherited homozygously since haplotype phasing could not be concluded. ii) Trio/duo analysis: In families for whom we had both parental DNA available (*n* = 81), we analyzed the family as a “trio” and if the DNA of only one parent was available (*n* = 32), we analyzed the family as a “duo”. By the “trio” and “duo” approach we looked for both homozygous and compound-heterozygous variants, and in trio families, we looked for non-inherited (*de novo*) variants as well. We then applied a homozygous recessive approach based on parental consanguinity and compared the detection rate of variants in novel genes between non-homozygous families (homozygosity-by-mapping <50 Mb) and homozygous families (homozygosity-by-mapping ≥50 Mb). If pathogenic variants in a single novel gene were detected using the approaches outlined above, we then considered it a potential novel candidate gene for SRNS (Fig. 1A and Table S7).

Of the 212 families with no molecular diagnosis, 45 were consanguineous families and 167 were non-consanguineous families. In the 45 consanguineous families, we found homozygously inherited variant in a single potential novel candidate gene in 18/33 (51.5%) singlets and 7/13 trios/duos (53.8%) (with 1 of 7 being *de novo* variant). In contrast, in 166 non-consanguineous families, we detected homozygously inherited variants in a single potential novel candidate gene in only 17/107 singlets (15.9%) (Fig. 1C). The rate of detection of candidate variants was significantly higher for trio/due analysis with 17/59 (28.8%) potential novel variants found (Fig. 1C). Of these, 9 were compound-heterozygously inherited, 2 were heterozygous *de novo*, and 6 were homozygously inherited variants. The *de novo* variants were detected in the genes *STRN*, *LMLN*, and *KCHN3*. None had been present in the gnomAD control population. None of these genes was reported to have a clinical phenotype in humans (not reported in OMIM) (Table S8).

In 12.5% of 320 families in this cohort (40/320) more than one likely deleterious variant was detected. These cases were deemed “inconclusive” (Fig. 1A). In 35.6% (114/320) of families, we did not detect a likely causative variant in any of the SRNS genes, phenocopy genes, or in any potential novel candidate gene (Fig. 1A).

In conclusion, the availability of parental DNA allows to discovery of a significantly higher number of novel candidate genes by examining compound heterozygous and *de novo* variants in outbred trios/duos as compared with singlets (Fig. 1C). Our current study showed similar detection rates of variants in genes known to be implicated in SRNS (27.5%), to earlier two studies (29.5% and 25%).^1^^,^^2^ As in our prior studies, we found a positive correlation between detection rate and consanguinity (Fig. 1B).^1^, ^2^, ^3^

Upon discovery, a rare variant in a novel gene is a theoretical candidate. Based on our prior experience, this initial family and variant become the basis for identifying additional variants in the candidate gene. With sufficient genetic evidence, functional studies are, then, warranted to support the biological impact of disease-causing variants. By this approach, we were able to functionally characterize 13 genes that were defined as potential candidate genes in a former exome sequencing study.^2^ These genes had been published as novel SRNS genes (Table S12). The current study has yielded three additional candidate genes, which have been further evaluated functionally and published as potential new SRNS-causing genes (*SYNPO2*, *TBC1D8B*, and *MYO9A*).

These discoveries not only broaden our understanding of the pathophysiology of SRNS but also have implications for clinical genetic testing. Specifically, providing an unequivocal molecular genetic diagnosis can preclude unnecessary diagnostic procedures, spare exposure to immunosuppressive agents with significant side effects, reveal tailored treatments (Alport/COQ) in select cases, suggest early screening for extra-renal diseases associated with these genetic conditions, and guide the identity of a living related donor in dominant diseases. Therefore, it remains essential to pursue the discovery of novel SRNS-associated genes including through trio exome sequencing as shown in this study.

## Ethics declaration

This study was approved by the institutional review board (IRB) of the University of Michigan (No. 2003-0116) and of Boston Children's Hospital (No. IRB-P00006200) and of institutions where families were recruited. Informed consent of each individual or its legal guardian was obtained.

## Author contributions

Conceptualization: R.S., S.Sh., F.H.; Data curation: R.S., S.Sh.; Formal analysis: R.S., S.Sh., F.B., K.D., K.Y., C.N.F., A.C.O., T.M.K., Y.M., V.K., M.Y.Z., K.L., A.J.M., B.M., K.S., S.Se., C.M.K., L.M.M., N.D.M., T.H., N.M., D.P., A.A.H., A.B., L.S., S.Sc., F.H.; Funding acquisition: F.H.; Investigation: R.S., S.Sh., F.B., K.D., K.Y., C.N.F., A.C.O., T.M.K., Y.M., V.K., M.Y.Z., K.L., A.J.M., B.M., K.S., S.Se., C.M.K., L.M.M., N.D.M., T.H., N.M., D.P., A.A.H., A.B., L.S., S.Sc., D.S., I.Z.B., I.S., L.A.E., H.H.A., M.A., N.A.S., M.M.N., J.A.K., S.E., M.A.S., H.M.F., S.M., R.P.L., M.J.G.S., F.H.; Methodology: S.M., R.P.L.; S.Sh.; Project administration: F.H.; Resources: I.Z.B., I.S., L.A.E., H.H.A., M.A., N.A.S., M.M.N., J.A.K., S.E., M.A.S., H.M.F., S.M., R.P.L., M.J.G.S., F.H; Software: S.Sh.; Supervision: F.H.; Validation: R.S.; Visualization: R.S.; Writing – original draft: R.S., F.H.; Writing – review & editing: S.Sh., F.H.

## Conflict of interests

The authors declared no conflict of interests.

## Funding

This research is supported by grants from the National Institutes of Health (NIH) (No. 5R01DK076683–13, RC2-DK122397 to F.H.). Sequencing and data processing were performed by the Yale Centers for Mendelian Genomics funded by the National Human Genome Research Institute (No. U54 HG006504). R.S. is supported by the Office of Faculty Development. K.Y., C.N.F., and N.D.M. are supported by an NIH Training Grant (No. 5T32DK007726) and by the 2017 Post-doctoral Fellowship Grant from the Harvard Stem Cell Institute. A.C.O. acknowledges support from the National Institutes of Health F32 Ruth L. Kirschstein Postdoctoral Individual National Research Service Award (No. DK122766). T.M.K. was supported by a Post-Doctoral Fellowship award from the KRESCENT Program, a national kidney research training partnership of the Kidney Foundation of Canada, the Canadian Society of Nephrology, and the Canadian Institutes of Health Research. V.K. is funded by the Deutsche Forschungsgemeinschaft grant (No. 403877094). M.Y.Z. was supported by a Fulbright Scholar fellowship (USEFP). K.L. is funded by the Deutsche Forschungsgemeinschaft Research Foundation (No. DFG461126211). A.J.M. is supported by the NIH (No. 5K12HD052896-13, 1K08DK125768-01A1), American Society of Nephrology Norman Siegel Research Scholar Grant, and Manton Center for Orphan Disease Research. S.Se. was supported by the Deutsche Forschungsgemeinschaft (German Research Foundation; No. 442070894). C.M.K. is supported by a grant from the Deutsche Forschungsgemeinschaft (German Research Foundation; No. KO 6579/2-1). N.M. is supported by funding from the National Institutes of Health (No. K08-DK127011). This research was also supported by the Jiang-Li Family Foundation.

## Data availability

The depersonalized data that this study is based on is available from the corresponding author upon request.
