# Supplementary material for: Expanding the spectrum of novel candidate genes using trio exome sequencing and identification of monogenic cause in 27.5% of 320 families with steroid-resistant nephrotic syndrome
Source: Genes Dis. 2024 Mar 28;12(2):101280. doi: 10.1016/j.gendis.2024.101280 (PMC11582537; doi:10.1016/j.gendis.2024.101280)
Supplement: Multimedia component 1 [file mmc1.pdf]

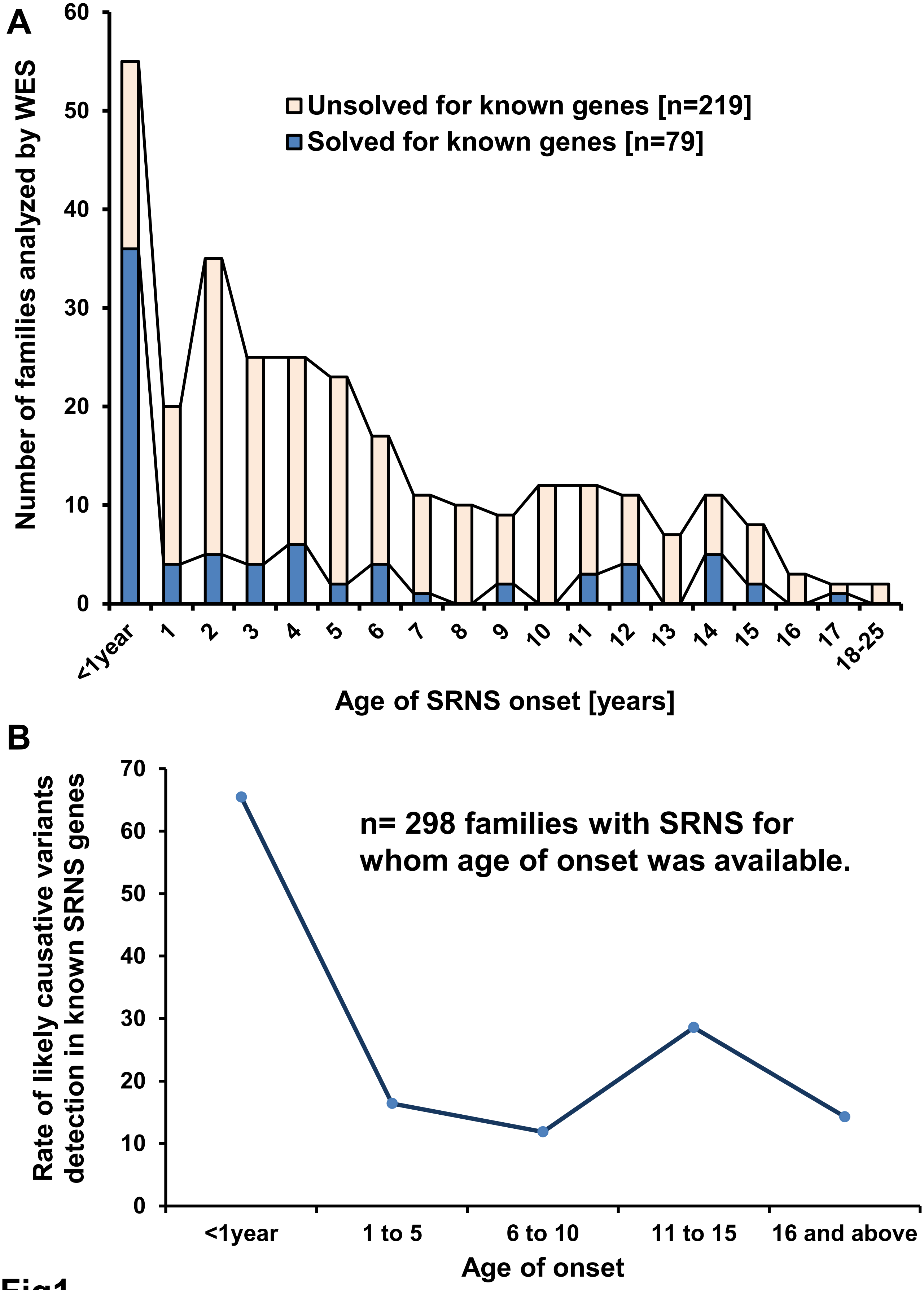
A
Number of families analyzed by WES
60
50
40
30
20
10
0
Unsolved for known genes [n=219]
Solved for known genes [n=79]
<1year
1
2
3
4
5
6
7
8
9
10
11
12
13
14
15
16
17
18-25
Age of SRNS onset [years]
B
Rate of likely causative variants detection in known SRNS genes
70
60
50
40
30
20
10
0
n= 298 families with SRNS for whom age of onset was available.
<1year
1 to 5
6 to 10
11 to 15
16 and above
Age of onset

sFig1

sFig1. Detection of likely causative variants in 27 known SRNS genes per age of SRNS onset.

Age of onset distribution (in years) for 298 families with SRNS.

The displayed 298 families represent the number of individuals with available data for the age of onset of nephrotic syndrome.

(A) Light orange histograms represent the number of families at each age of onset (years) of SRNS for 219 families without a molecular genetic diagnosis. Blue histograms show the number out of 79 families with causative variants identified for each age of onset.

(B) Graph indicates the percentage of pathogenic variant detection in known SRNS genes per range of age of SRNS onset.

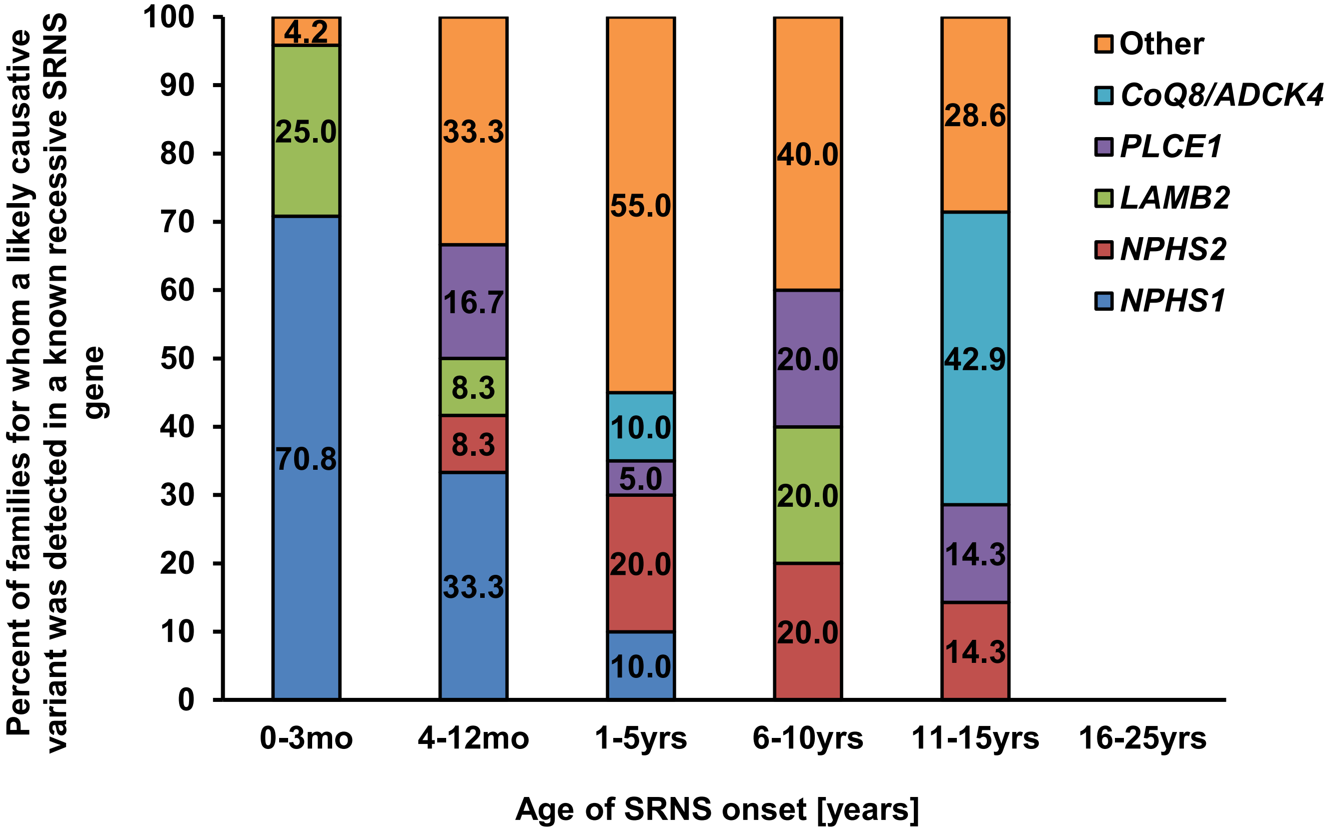

Percent of families for whom a likely causative variant was detected in a known recessive SRNS gene
100
90
80
70
60
50
40
30
20
10
0
4.2
25.0
70.8
33.3
16.7
8.3
8.3
33.3
55.0
10.0
5.0
20.0
10.0
40.0
20.0
20.0
20.0
28.6
42.9
14.3
14.3
Other
CoQ8/ADCK4
PLCE1
LAMB2
NPHS2
NPHS1
0-3mo
4-12mo
1-5yrs
6-10yrs
11-15yrs
16-25yrs
Age of SRNS onset [years]

sFig2

sFig2. Detection of likely causative variants in 21 recessive SRNS-causing genes in relation to the age of onset of proteinuria in clinically relevant age groups.

Percentage of families with likely causative variants detected per age group are shown, for 73 families for whom the age of onset SRNS data was available. Only the five most frequent recessive causes of childhood-onset SRNS in our cohort are included in the graph. Names of 17 other genes in which SRNS-causing variants were detected are grouped as ‘other’ (for a full see sTable 6). NPHS1 variants (blue) were frequently detected before age 12 months and were not found in patients older than 5 years. Data are not shown for families in whom data on the age of onset was not available. In families with >1 affected family member the mean age of onset from all affected individuals was calculated.
