# Supplementary material for: Expanding the spectrum of novel candidate genes using trio exome sequencing and identification of monogenic cause in 27.5% of 320 families with steroid-resistant nephrotic syndrome
Source: Genes Dis. 2024 Mar 28;12(2):101280. doi: 10.1016/j.gendis.2024.101280 (PMC11582537; doi:10.1016/j.gendis.2024.101280)
Supplement: Multimedia component 2 [file mmc2.pdf]

Supplementary Table 1: Inclusion and exclusion criteria for enrolment in study.

Inclusion Criteria	Exclusion Criteria
Age at onset under 25 years	Patients with non-nephrotic range proteinuria or hematuria only
AND	OR
Steroid resistant nephrotic syndrome	SSNS, acute GN (e.g. hypocomplementemia, gross hematuria), acute kidney injury.
AND/OR	OR
Steroid dependent nephrotic syndrome	Patient age >25 year at nephrotic syndrome onset
AND/OR	
FSGS or DMS	
OR	
Congenital/Infantile nephrotic syndrome	

DMS, diffuse mesangial sclerosis; **FSGS**, focal segmental glomerulosclerosis; **GN**, glomerulonephritis; **SSNS**, steroid sensitive nephrotic syndrome;

Supplementary Table 2: Age, sex, and ethnic characteristics of the steroid-resistant nephrotic syndrome cohort and of the individuals, in whom a likely causative monogenic variant was detected in either a 'steroid-resistant' nephrotic syndrome gene or a 'phenocopy gene'.

Demographics for age and sex are given for a subset of 100 individuals from 88 families, in whom a causative variant was detected in a SRNS gene or 21 individuals from 18 families in whom a causative variant was detected in a phenocopy gene are shown. Additionally, ethnicity data are given for all individuals in the cohort.

	Clinical characteristics of total cohort	Clinical characteristics of individuals with causative variant detected	
	Number of individuals (%)	Number of individuals with variant detected in SRNS genes (%)	Number of individuals with variant detected in phenocopy gene (%)
**Gender**			
Male	**192 (56%)**	**53 (53%)**	**14 (67%)**
Female	**145 (43%)**	**44 (44%)**	**7 (33%)**
Unknown	**6 (1.7%)**	**3 (3%)**	**0 (0%)**
Total	**343 (100%)**	**100 (100%)**	**21 (100%)**
**Median age (range) at diagnosis (in years)**	**4 (0-21)**	**2 (0-17)**	**5.5 (0-21)**
≤90 days	**44 (12.8%)**	**28 (28%)**	**1 (4.8%)**
>3m and ≤12m	**12 (3.5%)**	**10 (10%)**	**1 (4.8%)**
>1year and ≤7 years	**158 (46.1%)**	**29 (29%)**	**10 (47.6%)**
>7 years and ≤13 years	**68 (19.8%)**	**14 (14%)**	**3 (14.3%)**
> 13 years and ≤18 years	**35 (10.2%)**	**9 (9%)**	**4 (19%)**
> 18 years and ≤25 years	**3 (0.9%)**	**0 (0%)**	**1 (4.8%)**
not reported	**23 (6.7%)**	**10 (10%)**	**1 (4.8%)**
Total	**343 (100%)**	**100 (100%)**	**21 (100%)**
**Ethnicity**			
	Number of individuals (%)	Number of individuals (%)	Number of individuals (%)
Arabic	**125 (36.4%)**	**35 (35%)**	**9 (42.9%)**
Asian	**50 (14.6%)**	**21 (21%)**	**1 (4.8%)**
European/Caucasian	**73 (21.3%)**	**18 (18%)**	**6 (28.6%)**
Turkish	**33 (9.6%)**	**14 (14%)**	**0 (0%)**
Hispanic Latino	**13 (3.8%)**	**1 (1%)**	**3 (14.3%)**
Other	**33 (9.6%)**	**8 (8%)**	**1 (4.8%)**
Unknown/not indicated	**16 (4.7%)**	**3 (3%)**	**1 (4.8%)**
Total	**343 (100%)**	**100 (100%)**	**21 (100%)**

m, months; SRNS, steroid-resistant nephrotic syndrome.

Supplementary Table 3: 59 genes known to cause monogenic steroid-resistant nephrotic syndrome and mode of inheritance

Known SRNS Gene	Gene name	MOI	Reference
ACTN4	Actinin, alpha 4	AD	Kaplan *Nat Genet* 24(3):251, 2000
ADCK4 (COQ8B)	Co-enzyme Q8	AR	Ashraf *J Clin Invest* 123:5179, 2013
ANKFY1	Ankyrin Repeat And FYVE Domain Containing 1	AR	Hermle *J Am Soc Nephrol* 29:2123, 2018
ANLN	Actin-binding protein anillin	AD	Gbadegesin *J Am Soc Nephrol* 25:1991, 2014
ARHGAP24	Rho GTPase activating protein 24	AD	Akilesh *J Clin Invest* 121:4127, 2011
ARHGDIA	Rho GDP dissociation inhibitor (GDI) alpha	AR	Gee *J Clin Invest* 123:3243, 2013
AVIL	Advillin	AR	Rao *J Clin Invest* 127:4257, 2017
CD2AP	CD2 associated protein	AR	Lowik *Kiney Int* 10:1198, 2007
CDK20	Cyclin Dependent Kinase 20	AR	Ashraf *Nat Commun* 17:1960, 2018
COQ2	Coenzyme Q2 4-hydroxybenzoate polyprenyltransferase	AR	Diomedi-Camassei *J Am Soc Nephrol* 18:2773, 2007
COQ6	Coenzyme Q6 monooxygenase	AR	Heeringa *J Clin Invest* 121:2013, 2011
CRB2	Crumbs, Drosphilia, Homolog of 2	AR	Ebarasi *AJHG* 96: 153-161, 2015
CUBN	Cubilin (intrinsic factor-cobalamin receptor)	AR	Ovunc *J Am Soc Nephrol* 22:1815, 2011
DGKE	Diacylglycerol kinase epsilon	AR	Ozaltin *J Am Soc Nephrol* 24:377, 2013
DLC1	DLC1 Rho GTPase Activating Protein	AR	Ashraf *Nat Commun* 17:1960, 2018
EMP2	Epithelial membrane protein 2	AR	Gee *AJHG* 94:884, 2014
FAT1	Fat tumor suppressor, drosphila, homolog of, 1	AR	Gee *Nat Commun* 7:10822, 2016; Lahrouchi *Nat Commun* 10:1180, 2019

GAPVD1	GTPase Activating Protein And VPS9 Domains 1	AR	Hermle *J Am Soc Nephrol* 29:2123, 2018
GON7	GON7 Subunit Of KEOPS Complex	AR	Arrondel *Nat Commun* 10:3967, 2019
INF2	Inverted Formin 2	AD	Brown *Nat Genet* 42:72, 2010
ITGA3	Integrin, alpha 3 (antigen CD49C, alpha 3 subunit of VLA-3 receptor)	AR	Has *NEJM* 366:1508, 2012
ITGB4	Integrin, beta 4	AR	Kambham *AJKD* 36:190, 2000
ITSN1	Intersectin 1	AR	Ashraf *Nat Commun* 17:1960, 2018
ITSN2	Intersectin 2	AR	Ashraf *Nat Commun* 17:1960, 2018
KANK1	KN motif and ankyrin repeat domain-containing protein 1	AR	Gee *J Clin Invest* 125:2375, 2015
KANK2	KN motif and ankyrin repeat domain-containing protein 2	AR	Gee *J Clin Invest* 125:2375, 2015
KANK4	KN motif and ankyrin repeat domain-containing protein 3	AR	Gee *J Clin Invest* 125:2375, 2015
KIRREL1	Kirre Like Nephrin Family Adhesion Molecule 1	AR	Solanki *Kidney Int* 96:883, 2019
LAGE3	L antigen family member 3	AR	Braun *Nat Genet* 49:1529, 2017
LAMB2	Laminin, beta 2	AR	Zenker *Hum Mol Genet* 12:2625, 2004
LMX1B	LIM Homeobox Transcription Factor 1 Beta	AD	Dreyer *Nat Genet* 19:47 1998
MAGI2	Membrane-associated guanylate kinase, WW and PDZ domains-containing 2	AR	Bierzynska *J Am Soc Nephrol* 28:1614, 2017
MYH9	Myosin heavy chain 9, non-muscle	AD	Heath *AJHG* 69:1033, 2001
MYO1E	Homo sapiens myosin IE (MYO1E)	AR	Mele *NEJM* 365:295, 2011
NPHS1	Nephrin	AR	Kestila *Mol Cell* 1:575, 1998
NPHS2	Podocin	AR	Boute *Nat Genet* 24:349, 2000
NUP85	Nucleoporin 85-KD	AR	Braun *Nat Genet* 48:457, 2016
NUP93	Nucleoporin, 93-KD	AR	Braun *Nat Genet* 48:457, 2016
NUP107	Nucleoporin, 107-KD	AR	Miyake *AJHG* 97:555, 2015
NUP133	Nucleoporin 133-KD	AR	Braun *Nat Gene* 48:457, 2016
NUP205	Nucleoporin, 205-KD	AR	Braun *Nat Genet* 48:457, 2016
OSGEP	O-sialoglycoprotein endopeptidase	AR	Braun *Nat Genet* 49:1529, 2017

PAX2	Paired Box 2	AD	Barua *J Am Soc Nephrol* 25:1942, 2014
PDSS2	Prenyl (decaprenyl) diphosphate synthase, subunit 2	AR	Lopez *AJHG* 79:1125, 2006
PLCE1	Phospholipase C, epsilon 1	AR	Hinkes *Nat Genet* 38:1397, 2006
PTPRO	Protein tyrosine phosphatase, receptor type, O	AR	Ozaltin *AJHG* 89:139, 2011
SCARB2	Scavenger receptor class B, member 2	AR	Badhwar *Brain* 127: 2173, 2004<br>Berkovic Am J Hum Genet, 2008
SGPL1	Sphingosine 1 phosphate lyase 1	AR	Lovric *J Clin Invest* 127: 912, 2017
SMARCAL1	SWI/SNF related, matrix associated, actin dependent regulator<br>of chromatin, subfamily a-like 1	AR	Boerkoel *Nat Genet* 30:215, 2002
TNS2	Tensin 2	AR	Ashraf *Nat Commun* 17:1960, 2018
TP53RK	TP53-regulating kinase	AR	Braun *Nat Genet* 49:1529, 2017
TPRKB	TP53RK binding protein	AR	Braun *Nat Genet* 49:1529, 2017
TRPC6	Transient receptor potential cation channel, subfamily C, member 6	AD	Winn *Science* 308:1801, 2005
WDR4	WD Repeat Domain 4	AR	Braun *Am J Med Gene*t *A* 176:2460, 2018
WDR73	WD repeat-containing protein 73	AR	Colin *AJHG* 95:637, 2014
WT1	Wilms Tumor 1	AD	Jeanpierre *Am J Hum Gene*t 62:824, 1998
XPO5	Exportin 5	AR	Braun *Nat Genet* 48:457, 2016
YRDC	YrdC N6-Threonylcarbamoyltransferase Domain Containing	AR	Arrondel *Nat Commun* 10:3967, 2019

AD, autosomal dominant; **AR**, autosomal recessive, **MOI**, mode of inheritance; **XLR**, X-linked recessive.

Supplementary Table 4. Variant filtering process for identification of disease-causing variants.

Basic assumptions: 1) Defined clinical phenotype. 2) Known genes with similar phenotype have been excluded. 3) “Variant” implies that an allele changes the phenotype. 4) Full (autosomal recessive, **AR**) or incomplete (autosomal dominant, **AD**) penetrance (age related).

- **Include allele as likely disease causing if:**
  - Truncating mutation (stop, abrogation of start or stop, obligatory splice, frame-shift) in an expressed gene (well annotated mRNA, conservation, protein expression) or:
  - Missense mutation if:
    - Continuously conserved at least up to *Danio rerio (*vertebral or beyond). AND:
    - Loss of function in human allele is supported by functional data and:
    - Allele segregates with the affected status in the family.
    - Genotype- phenotype correlation

- **Exclude allele as disease causing if:**
  - Heterozygous allele frequency >1% (for AR) or >0.1% (for AD) if reported homozygous (>3) in gnomAD in AR disease, or frequent heterozygously (>20) in AD disease. *Exception: R229Q variant of *NPHS2*.
  - AR: Non-segregation (e.g. both alleles in cis; affected family member is without the variant; unaffected parent is with homozygous variant).
  - AD: Non segregation (e.g. affected family member does not carry the variant; but if an unaffected family member carries the allele consider incomplete penetrance and variable expressivity).

AD, autosomal dominant; AR, autosomal recessive; gnomAD, The Genome Aggregation Database

Supplementary Table 5: Summary of likely causative variants detected in one of 6 steroid-resistant nephrotic syndrome phenocopy genes in 18 of 320 families with steroid-resistant nephrotic syndrome by family and clinical phenotype.

Family	Gene	Individual	Nt change c.	AA change p.	Zygosity	Cons.	SIFT	Mut.Taster	PP2	gnomAD	Clinical	Biopsy	Age (years)	Sex	Ethnicity	Syndromic	Biobase	ACMG
B3782	*ATP6V0A4*	B3782_21	c.710_712del	p.Lys237del	Hom	N/A	N/A	N/A	N/A	0	INS	n/a	4-12 m	M	Arabic	No	Yes	VUS
B1523	*COL4A3*	B1523_21	c.820_821delinsC	p.Gly274Hisfs*49	Hom	N/A	N/A	N/A	N/A	n/p	SRNS	MP	4	F	Arabic	No	Yes	LP
B2356	*COL4A3*	B2356_21	c.1354G>A	p.Gly452Arg	Het	*D.m*	D	DC	D	0	SRNS	FSGS	20	F	Jewish (Moroccan)	Deafness	No	P
B2735	*COL4A3*	B2735_21	c.2417dup	p.Gly807Argfs*28	Hom	N/A	N/A	N/A	N/A	n/p	SRNS	MP	13	M	Arabic	Deafness	Yes	P
B3349	*COL4A3*	B3349_21	c.4421T>C; c.1504+1G>A	p.Leu1474Pro; SPLICE	Comp. Het	*D.m;* N/A	D; N/A	DC; N/A	D; N/A	0; n/p	SRNS	FSGS	12	M	Jewish (Ashkenazi)	No	Yes; Yes	P; P
	COL4A3	B3349_22									SRNS	n/a	8	M		No		
A3581	*COL4A3*	A3581_21	c.2717dup	p.Gly907Trpfs*33	Hom	N/A	N/A	N/A	N/A	n/p	SRNS	MP	5	F	Arabic	No	No	LP
F1177	*COL4A4*	F1177_21	c.1389del	p.Asn464Thrfs*7	Het	N/A	N/A	N/A	N/A	n/p	SRNS	MP	6	M	n/a	Deafness	No	P
B1743	*COL4A4*	B1743_21	c.914_930+29 del	SPLICE	Hom	N/A	N/A	N/A	N/A	n/p	SRNS	Alport	n/a	M	Arabic	No	Yes	P
B3487	*COL4A4*	B3487_21	c.2626G>A	p.Gly876Arg	Hom	*D.r*	D	DC	D	n/p	SRNS	n/a	6	M	Asian	No	Yes	LP
A965	*COL4A5*	A965_21	c.276_276+5del	p.Gly93Valfs*62	Hemi	N/A	N/A	N/A	N/A	n/p	SRNS	FSGS	15	M	Eu/C	No	Yes	P
B1235	*COL4A5*	B1235_21	c.4309C>G	p.Gln1437Glu	Het	*D.r*	D	DC	PD	1	SDNS	C1qN	2	F	Arabic	No	No	LB
B2535	*COL4A5*	B2535_21	c.3095T>C	p.Met1032Thr	Hemi	*D.r*	T	DC	PD	0	SRNS	MP	12	M	Hispanic	Intellectual disability	No	VUS
B3610	*COL4A5*	B3610_21	c.142-1G>A	SPLICE	Hemi	N/A	N/A	N/A	N/A	n/p	Alport	n/a	0-3 m	M	Eu/C	No	No	P
B3881	*COL4A5*	B3881_21	c.2178A>T	p.Glu726Asp	Hemi	*D.m*	D	DC	D	n/p	SRNS	FSGS	16	M	Hispanic	No	No	LB
	COL4A5	B3881_22									SRNS	FSGS	4	F		No		
A1760	*DGKE*	A1760_21	c.1009C>T	p.Arg337*	Hom	N/A	N/A	N/A	N/A	0	pSSNS	n/a	4	M	Arabic	No	Yes	P
A2476	*FN1*	A2476_21	c.984_989del	p.Asn328_Gln330 delinsLys	Het	N/A	N/A	N/A	N/A	n/p	SRNS	MP	5	F	Arabic	No	No	LP
B3359	*FN1*	B3359_21	c.4352C>T	p.Ser1451Phe	Het	*X.t*	D	N/A	D	0	SDNS	n/a	3	F	Eu/C	No	No	VUS
B1119	*WDR19*	B1119_21	c.3533G>A	p.Arg1178Gln	Hom	*C.i*	T	DC	D	0	Proteinuria	DMS	1.5	M	Eu/C	Blindness	No	LP

AA, amino acid; **ACMG**, American College of Medical Genetics; **Ce**, Caenorhabditis elegans; **CNS**, congenital nephrotic syndrome; **Comp. Het**, compound heterozygous; **Cons.**, conservation; **Ci**, Ciona Intestinalis; **C1qN**, C1q nephropathy, **D**, deleterious; **DC**, disease causing; **del**, deletion; **DMS**, diffuse mesangial sclerosis; **Dr**, drosophila melanogaster; **dup**, duplication; **ESRD**, end-stage renal disease; **Eu/C**, European/Caucasian; **F**, female; **fs**, frameshift; **FSGS**, focal segmental glomerulosclerosis; **Gg**, Gallus Gallus; **GGS**, global glomerulosclerosis; **gnomAD**, Genome Aggregation Database; **Het**, heterozygous; **Hom**, homozygous; **IF**, interstitial nephritis; **INS**, infantile nephrotic syndrome; **LB**, likely benign; **LP**, likely pathogenic; ;**m**, months; **M**, male; **MP**, mesangial proliferation; **Mut. Taster**, mutation taster; **n/a**, not applicable; **Nt.**, nucleotide; **P**, pathogenic; **PM**, polymorphism; **PP2**, PolyPhen-2; **pSSNS**, partial steroid sensitive nephrotic syndrome; **SDNS**, steroid dependent nephrotic syndrome; **SIFT**, sorting intolerant from tolerant; **SRNS**, steroid resistant nephrotic syndrome; **T**, tolerated; **UTI**, urinary tract infection; **VUS**, variant of unknown significance; **Xt**, Xenopus Tropicalis.

Supplementary Table 6: Summary of 108 likely causative variants detected in one of 27 SRNS causing genes in 88 families (98 individuals) of 320 families with SRNS by individual, and clinical phenotype.

Gene	Individual	Nt change c.	AA change p.	Zygosity	Cons.	SIFT	Mut. Taster	PP2	gnomAD	Clinical	Biopsy	Age (years)	Sex	Ethnicity	Syndromic	Family	Biobase	ACMG
ACTN4	A67_21	c.1117A>G	p.Met373Val	Het	*Ci*	D	DC	0	n/p	SRNS	FSGS	14	M	African/African-American	No	A67	Yes	VUS/LP
ACTN4	B3954_21	c.1940A>G	p.Asn647Ser	Het	*Gg*	D	DC	1	n/p	SDNS	n/a	11	M	Indian	No	B3954	No	VUS/LP
ADCK4/COQ8B	A3987_21	c.1339dup	p.Glu447Glyfs*10	Hom	*Xt*	n/a	n/a	n/a	0/6/242266	SRNS	n/a	n/a	F	n/a	Deafness	A3987	Yes	P
ADCK4/COQ8B	A3987_22	c.1339dup	p.Glu447Glyfs*10	Hom	*Xt*	n/a	n/a	n/a	0/6/242266	SRNS	n/a	n/a	M	n/a	No	A3987	Yes	P
ADCK4/COQ8B	B1744_21	c.289G>A	p.Gly97Arg	Hom	*Dm*	n/a	n/a	0.96	0/1/31358	n/a	FSGS	4	F	Eu/C	No	B1744	No	P
ADCK4/COQ8B	A2338_21	c.532C>T	p.Arg178Trp	Hom	*Dr*	n/a	n/a	0.99	0/7/279154	SRNS	FSGS	n/a	F	n/a	No	A2338	Yes	VUS/LP
ADCK4/COQ8B	A2338_22	c.532C>T	p.Arg178Trp	Hom	*Dr*	n/a	n/a	0.99	0/7/279154	SRNS	FSGS	14	M	n/a	No	A2338	Yes	VUS/LP
ADCK4/COQ8B	A60_21	c.101G>A	p.Trp34*	Hom	*n/a*	n/a	n/a	n/a	n/p	SRNS	FSGS	3	M	n/a	No	A60	Yes	P
ADCK4/COQ8B	B3278_21	c.1199dup	p.His400Glnfs*11	Hom	*n/a*	n/a	n/a	n/a	0/1/251484	Proteinuria	FSGS	11	F	Turkish	No	B3278	Yes	P
ADCK4/COQ8B	B3298_21	c.532C>T	p.Arg178Trp	Hom	*Sc*	D	DC	0.99	0/7/279154	SRNS	FSGS	11	M	Eu/C	No	B3298	Yes	VUS/LP
COQ2	A593_21	c.376C>G	p.Arg126Gly	Comp. Het	*Dr*	D	DC	0.3	0/2/172898	SRNS	DMS	2	F	n/a	Intellectual disability	A593	Yes	LP
COQ2	A593_21	c.288dup	p.Ala97Argfs*56	Comp. Het	*n/a*	D	DC	0.2	0/30/99652							A593	Yes	LP
COQ2	A593_22	c.376C>G	p.Arg126Gly	Comp. Het	*Dr*	D	DC	0.3	0/2/172898	SRNS	DMS	2	M	n/a	Short stature	A593	Yes	LP
COQ2	A593_22	c.288dup	p.Ala97Argfs*56	Comp. Het	*n/a*	D	DC	0.2	0/30/99652							A593	Yes	LP
COQ2	A593_23	c.376C>G	p.Arg126Gly	Comp. Het	*Dr*	D	DC	0.3	0/2/172898	SRNS	DMS	2	M	n/a	n/a	A593	Yes	LP
COQ2	A593_23	c.288dup	p.Ala97Argfs*56	Comp. Het	*n/a*	D	DC	0.2	0/30/99652							A593	Yes	LP
COQ2	B3454_21	c.890A>G	p.Tyr297Cys	Hom	*Sc*	D	DC	0.93	0/1/246202	INS	n/a	4-12 m	M	Eu/C	No	B3454	Yes	LP
COQ2	B3822_21	c.571G>C	p.Val191Leu	Comp. Het	*Sc*	D	DC	1	n/p	SRNS	DMS	1	F	Arabic	Yes	B3822	No	P
COQ2	B3822_21	c.288dup	p.Ala97Argfs*56	Comp. Het	*n/a*	D	DC	1	0/30/101658							B3822	Yes	P
CRB2	B3540_21	c.3214C>T	p.Arg1072Cys	Comp. Het	*Gg*	D	DC	0.7	0/2/208848	SRNS	n/a	4	F	Arabic	No	B3540	No	P
CRB2	B3540_21	c.1827C>A	p.Cys609*	Comp. Het	*n/a*	D	DC	0.7	0/1/248408							B3540	No	P
CRB2	A3981_24	c.3767G>A	p.Gly1256Asp	Hom	*Ci*	D	DC	0.99	n/p	INS	MCNS	4-12 m	M	n/a	Bicuspid aorta	A3981	No	VUS/LP

Supplementary table 6, cont.

Gene	Individual	Nt change c.	AA change p.	Zygosity	Cons.	SIFT	Mut. Taster	PP2	gnomAD	Clinical	Biopsy	Age (years)	Sex	Ethnicity	Syndromic	Family	Biobase	ACMG
FAT1	B3819_21	c.4097C>T	p.Thr1366Ile	Comp. Het	*Gg*	D	DC	0.9	0/1/31410	SRNS	n/a	8	F	Arabic	No	B3819	No	VUS
FAT1	B3819_21	c.6302G>A	p.Arg2101His	Comp. Het	*Xt*	D	DC	0.9	0/7/280582							B3819	No	VUS
INF2	F1332_21	c.170T>G	p.Leu57Arg	Het	*Dr*	D	DC	1	n/p	SRNS	FSGS	15	F	Arabic	Intellectual disability	F1332	Yes	LP
ITSN2	B3484_21	c.1313A>G	p.Glu438Gly	Comp. Het	*Dm*	D	DC	0.99	3/154/250082	SRNS	n/a	4.5	M	Asian	No	B3484	Yes	LB
ITSN2	B3484_21	c.796T>A	p.Phe266Ile	Comp. Het	*Gg*	D	DC	0.99	0/3/251316							B3484	No	LB
KANK1	B3453_21	c.3382C>A	p.Gln1128Lys	Comp. Het	*Ci*	T	DC	0.8	0/20/251234	CNS	n/a	0-3 m	M	Eu/C	No	B3453	No	Benign
KANK1	B3453_21	c.3619G>C	p.Ala1207Pro	Comp. Het	*Dr*	T	DC	0.8	0/16/282820							B3453	No	Benign
LAGE3	F1081_21	c.355T>C	p.Ser119Pro	Hemi	*Dr*	T	PM	0.98	n/p	SRNS	FSGS	4	F	Arabic	No	F1081	No	VUS
LAMB2	B2428_22	c.5368C>T	p.Gln1790*	Hom	*n/a*	n/a	n/a	n/a	n/p	CNS	n/a	0-3 m	F	Turkish	No	B2428	No	LP
LAMB2	F1234_21	c.4140C>A	p.Asn1380Lys	Comp. Het	*Dr*	D	DC	0	0/74/282864	INS	n/a	4-12 m	F	Arabic	No	F1234	No	VUS/ P
LAMB2	F1234_21	c.4177C>T	p.Leu1393Phe	Comp. Het	*Dr*	D	DC	0	0/1/251374							F1234	Yes	VUS/ P
LAMB2	F1200_21	c.4573+1G>A	obligatory splice site - 100%	Hom	*n/a*	n/a	n/a	n/a	n/p	SRNS	n/a	n/a	F	Arabic	No	F1200	Yes	P
LAMB2	B2646_21	c.5182C>T	p.Gln1728*	Hom	*n/a*	n/a	n/a	n/a	0/1/251432	CNS	n/a	0-3 m	F	Turkish	Eye anomalies	B2646	Yes	P
LAMB2	B2669_21	c.1890G>C	p.Gln630His	Hom	*Dr*	D	DC	1	n/p	CNS	n/a	0-3 m	F	Turkish	Bullosa	B2669	Yes	P
LAMB2	B3240_21	c.3797+4C>T	splice site	Comp. Het	*n/a*	n/a	n/a	n/a	0/68/281856	SRNS	n/a	6	F	Turkish	No	B3240	No	VUS
LAMB2	B3240_21	c.2669C>T	p.Thr890Ile	Comp. Het	*Dr*	n/a	n/a	n/a	0/192/251278							B3240	Yes	VUS
LAMB2	B3463_21	c.234del	p.Ile78Metfs*34	Comp. Het	*n/a*	n/a	n/a	n/a	n/p	CNS	n/a	0-3 m	M	Eu/C	No	B3463	No	P
LAMB2	B3463_21	c.825T>A	p.Tyr275*	Comp. Het	*n/a*	n/a	n/a	n/a	0/1/249606							B3463	Yes	P
LAMB2	A4938_25	c.253delG	p.Glu85Lysfs*27	Hom	*n/a*	n/a	n/a	n/a	n/p	CNS	n/a	0-3 m	M	n/a	No	A4938	Yes	P
LAMB2	B4015_21	c.4667C>T	p.Ala1556Val	Comp. Het	*Dr*	D	DC	0.99	1/73/282316	SRNS	n/a	9	M	Indian	No	B4015	Yes	P
LAMB2	B4015_21	c.5182C>T	p.Gln1728*	Comp. Het	*n/a*	D	DC	0.99	0/1/251432							B4015	Yes	P
LAMB2	B3932_21	c.1931_1938del	p.Arg644Profs*16	Hom	*n/a*	n/a	n/a	n/a	0/2/251310	CNS	n/a	0-3 m	M	Indian	No	B3932	No	P
LAMC1	A3153_22	c.1468C>T	p.Arg490Trp	Comp. Het	*Dr*	T	PM	0.6	0/144/282836	SRNS	FSGS	3	M	n/a	No	A3153	No	VUS
LAMC1	A3153_22	c.3631G>A	p.Ala1211Thr	Comp. Het	*Dm*	T	PM	0.6	0/50/277102							A3153	No	VUS

Supplementary table 6, cont.

Gene	Individual	Nt change c.	AA change p.	Zygosity	Cons.	SIFT	Mut. Taster	PP2	gnomAD	Clinical	Biopsy	Age (years)	Sex	Ethnicity	Syndromic	Family	Biobase	ACMG
LMX1B	A2157_21	c.685C>A	p.Gln229Lys	Het	*Dr*	D	DC	0.99	n/p	SRNS	MP	2	M	n/a	No	A2157	No	LP
MYH9	B4116_21	c.3022C>G	p.Leu1008Val	Het	*Dm*	D	n/a	0.3	0/7/251492	SRNS	FSGS	12	F	Arabic	Seizures	B4116	No	VUS
MYO1E	B1731_21	c.976C>T	p.Arg326Trp	Hom	*Sc*	D	DC	1	1/110/282840	INS	MCNS	4-12 m	M	Eu/C	No	B1731	No	VUS
NPHS1	A3598_21	c.515_517del	p.Thr172del	Hom	*n/a*	n/a	n/a	n/a	0/2/251116	SRNS	IN	1.5	M	n/a	Septal defect	A3598	Yes	P
NPHS1	B1814_23	c.2206G>T	p.Val736Leu	Comp. Het	*Dm*	D	DC	0.2	n/p	n/a	n/a	n/a	F	Eu/C	No	B1814	Yes	VUS/LP
NPHS1	B1814_23	c.1757+1G>A	splice	Comp. Het	*n/a*	D	DC	0.2	n/p							B1814	No	VUS/LP
NPHS1	A2232_21	c.1570G>A	p.Ala524Thr	Comp. Het	*Dm*	D	DC	1	n/p	SRNS	FSGS	5	M	n/a	PS	A2232	Yes	P
NPHS1	A2232_21	c.1701C>A	p.Cys567*	Comp. Het	*n/a*	D	DC	1	0/10/276640							A2232	Yes	P
NPHS1	B2441_21	c.1099C>T	p.Arg367Cys	Comp. Het	*Gg*	D	DC	0.96	0/10/251356	CNS	FSGS	0-3 m	F	Turkish	mild developmental delay	B2441	Yes	P
NPHS1	B2441_21	c.2152C>T	p.Gln718*	Comp. Het	*n/a*	D	DC	0.96	n/p							B2441	No	P
NPHS1	B2441_22	c.1099C>T	p.Arg367Cys	Comp. Het	*Gg*	D	DC	0.96	0/10/251356	CNS	FSGS	0-3 m	M	Turkish	Microcephaly, Mental retardation, seizures	B2441	Yes	P
NPHS1	B2441_22	c.2152C>T	p.Gln718*	Comp. Het	*n/a*	D	DC	0.96	n/p							B2441	No	P
NPHS1	B3172_21	c.1235G>T	p.Gly412Val	Hom	*Dr*	D	DC	0.99	n/p	INS	n/a	4-12 m	F	Turkish	No	B3172	No	LP
NPHS1	B3277_21	c.3478C>T	p.Arg1160*	Hom	*n/a*	n/a	n/a	n/a	0/25/251434	CNS	n/a	0-3 m	F	Turkish	PFO	B3277	Yes	P
NPHS1	B3452_21	c.794G>C	p.Cys265Ser	Hom	*Dm*	D	DC	1	0/7/249322	CNS	n/a	0-3 m	M	Eu/C	No	B3452	No	LP
NPHS1	B3455_21	c.3027C>G	p.Tyr1009*	Hom	*n/a*	n/a	n/a	n/a	0/2/251448	CNS	n/a	0-3 m	M	Eu/C	No	B3455	Yes	P
NPHS1	B3456_21	c.1099C>T	p.Arg367Cys	Hom	*n/a*	D	DC	1	0/10/251356	CNS	n/a	0-3 m	M	Eu/C	No	B3456	Yes	P
NPHS1	B3458_21	c.614_621delinsTT	p.Thr205Lysfs*4	Hom	*n/a*	n/a	n/a	n/a	0/5/ 251268	CNS	n/a	0-3 m	M	Eu/C	No	B3458	Yes	P
NPHS1	B3458_22	c.614_621delinsTT	p.Thr205Lysfs*4	Hom	*n/a*	n/a	n/a	n/a	0/5/ 251268	CNS	n/a	n/a	n/a	Eu/C	n/a	B3458	Yes	P
NPHS1	B3459_21	c.2600G>A	p.Gly867Asp	Hom	*Dr*	D	DC	0.99	0/4/239748	CNS	n/a	0-3 m	M	Eu/C	No	B3459	Yes	VUS/LP
NPHS1	B3460_21	c.1758-8_1785del	splice	Hom	*n/a*	n/a	n/a	n/a	0/1/235108	CNS	n/a	0-3 m	M	Eu/C	No	B3460	Yes	P
NPHS1	B3462_21	c.3478C>T	p.Arg1160*	Hom	*n/a*	n/a	n/a	n/a	0/25/251434	CNS	n/a	0-3 m	M	Eu/C	No	B3462	Yes	P
NPHS1	B3613_21	c.3250dupG	p.Val1084Glyfs*12	Hom	*n/a*	n/a	n/a	n/a	0/18/236910	INS	n/a	4-12 m	F	Arabic	No	B3613	Yes	P
NPHS1	A5128_22	c.1219C>T	p.Arg407Trp	Hom	*Mm*	D	PM	0.98	0/6/282766	CNS	n/a	0-3 m	M	n/a	No	A5128	Yes	P
NPHS1	B3854_22	c.1006G>C	p.Gly347Glu	Hom	*Dm*	D	DC	0.99	n/p	INS	n/a	4-12 m	F	Arabic	GDD	B3854	Yes	VUS/LP

Supplementary table 6, cont.

Gene	Individual	Nt change c.	AA change p.	Zygosity	Cons.	SIFT	Mut. Taster	PP2	gnomAD	Clinical	Biopsy	Age (years)	Sex	Ethnicity	Syndromic	Family	Biobase	ACMG
NPHS1	B4066_21	c.1888delC	p.Leu630fs	Hom	*n/a*	T	PM	P0.83	n/p	CNS	n/a	0-3 m	M	Indian	No	B4066	No	P
NPHS1	B4120_21	c.1379G>A	p.Arg460Gln	Hom	*n/a*	T	PM	0.5	0/1/250476	CNS	n/a	0-3 m	F	Arabic	No	B4120	Yes	P
NPHS1	B3807_21	c.802C>T	p.Arg268*	Hom	*n/a*	n/a	n/a	n/a	0/8/280584	CNS	n/a	0-3 m	F	Arabic	No	B3807	Yes	P
NPHS1	B2607_21	c.809G>T	p.Gly270Val	Hom	*Dm*	D	DC	1	n/p	CNS	n/a	0-3 m	F	Turkish	No	B2607	No	LP
NPHS1	F626_21	c.515_517del	p.Thr172del	Hom	*n/a*	n/a	n/a	n/a	0/2/251116	CNS	MP	0-3 m	F	Arabic	No	F626	Yes	LP
NPHS1	B3040_21	c.1099C>T	p.Arg367Cys	Hom	*n/a*	D	DC	0.96	0/10/251356	CNS	n/a	0-3 m	F	Turkish	No	B3040	Yes	P
NPHS2	B1537_22	c.686G>A	p.Arg229Gln	Comp. Het	*Dm*	T	PM	0	69/3526/119108	SRNS	MCNS	4	M	Eu/C	No	B1537	Yes	P
NPHS2	B1537_22	c.851C>T	p.Ala284Val	Comp. Het	*Dm*	T	PM	0	0/1/249998							B1537	Yes	P
NPHS2	B1537_21	c.686G>A	p.Arg229Gln	Comp. Het	*Dm*	T	PM	0	69/3526/119108	SRNS	MCNS	19 m	M	Eu/C	No	B1537	Yes	P
NPHS2	B1537_21	c.851C>T	p.Ala284Val	Comp. Het	*Dm*	T	PM	0	0/1/249998							B1537	Yes	P
NPHS2	B1768_21b	c.371G>A	p.Cys124Tyr	Hom	*Dm*	D	DC	0.99	n/p	INS	FSGS	4-12 m	F	Eu/C	No	B1768	Yes	LP
NPHS2	A1248_21	c.1A>T	start loss	Hom	*n/a*	n/a	n/a	n/a	n/p	SRNS	FSGS	6	M	n/a	No	A1248	Yes	P
NPHS2	B2605_21	c.378G>C	p.Lys126Asn	Hom	*Dm*	D	DC	0.99	n/p	SRNS	n/a	4	F	Turkish	Klippel-Trenaunay syndrome	B2605	Yes	P
NPHS2	B3466_21	c.378G>C	p.Lys126Asn	Hom	*Dm*	D	DC	0.99	n/p	SRNS	n/a	6	M	Eu/C	No	B3466	Yes	P
NPHS2	B3472_21	c.378G>C	p.Lys126Asn	Hom	*Dm*	D	DC	0.99	n/p	SRNS	n/a	1	F	Asian	No	B3472	Yes	P
NPHS2	B3584_21	c.524C>T	p.Pro175Leu	Hom	*Dr*	D	DC	0.99	0/1/ 214156	SRNS	n/a	14	M	Arabic	No	B3584	No	LP
NPHS2	B3584_22	c.524C>T	p.Pro175Leu	Hom	*Dr*	D	DC	0.99	0/1/ 214156	SRNS	n/a	n/a	F	Arabic	No	B3584	No	LP
NPHS2	B3704_21	c.502C>T	p.Arg168Cys	Hom	*Dr*	D	DC	1	0/0/221518	SRNS	n/a	n/a	F	Arabic	PS	B3704	Yes	P
NPHS2	B3942_21	c.502C>T	p.Arg168Cys	Hom	*Sc*	D	DC	1	0/0/221518	Proteinuria	n/a	5	M	Indian	No	B3942	Yes	P
NUP107	A1663_21	c.303G>A	p.Met101Ile	Comp. Het	*Dr*	D	DC	0	0/1/250664	SRNS	MCNS	2	M	n/a	No	A1663	Yes	VUS
NUP107	A1663_21	c.91A>G	p.Arg31Gly	Comp. Het	*Xt*	D	DC	0	1/276/281216							A1663	No	VUS
NUP107	A1830_21	c.2129_2131del	p.Glu710del	Comp. Het	*n/a*	n/a	n/a	n/a	-	ESRD	GGS; IF	5	M	n/a	Developmental delay; microcephaly	A1830	Yes	P
NUP107	A1830_21	c.1021dup	p.Glu341Glyfs*3	Comp. Het	*n/a*	n/a	n/a	n/a	n/p							A1830	Yes	P
NUP93	A4410_21	c.1463A>G	p.His488Arg	Hom	*Dm*	T	DC	0.99	n/p	SRNS	n/a	2	M	n/a	Propionic acidemia	A4410	No	LP
NUP93	B3596_21	c.1162C>T	p.Arg388Trp	Hom	*Ce*	D	DC	0.94	1/221/282730	SRNS	FSGS	5	F	Arabic	No	B3596	Yes	VUS/LP

Supplementary table 6, cont.

Gene	Individual	Nt change c.	AA change p.	Zygosity	Cons.	SIFT	Mut. Taster	PP2	gnomAD	Clinical	Biopsy	Age (years)	Sex	Ethnicity	Syndromic	Family	Biobase	ACMG
OSGEP	B1092_21	c.775A>T	p.Ile259Phe	Hom	*Sc*	D	DC	0.93	n/p	INS	n/a	4-12 m	M	African/ African-American	microcephaly	B1092	No	LP
PAX2	B3428_21	c.76dup	p.Val26Glyfs*28	Het	*n/a*	n/a	n/a	n/a	n/p	NS	n/a	16	F	Arabic	No	B3428	No	P
PAX2	B3428_22	c.76dup	p.Val26Glyfs*28	Het	*n/a*	n/a	n/a	n/a	n/p	NS	n/a	14	F	Arabic	No	B3428	No	P
PLCE1	B3281_21	c.4600A>G	p.Lys1534Glu	Hom	*Dr*	D	DC	0.96	n/p	INS	n/a	4-12 m	F	Turkish	No	B3281	Yes	VUS/ LP
PLCE1	A4622_21	del of Exon 2-20	-	Hom	*n/a*	n/a	n/a	n/a	n/p	INS	FSGS	4-12 m	M	n/a	No	A4622	No	P
PLCE1	A4939_23	c.5951_5953del	p.Asn1984del	Hom	*n/a*	n/a	n/a	n/a	n/p	SRNS	n/a	2	M	n/a	No	A4939	Yes	VUS/ LP
PLCE1	A4997_22	c.4451C>T	p.Ser1484Leu	Hom	*Ce*	D	DC	1	0/1/249446	pSSNS	FSGS	6	M	n/a	Intelectual disability	A4997	Yes	VUS/ LP
PLCE1	B3820_23	c.1477C>T	p.Arg493*	Hom	*n/a*	n/a	n/a	n/a	0/3/249224	SRNS	n/a	n/a	F	Arabic	No	B3820	Yes	P
PLCE1	B2484_21	c.1081T>C	p.Trp361Arg	Hom	*Dr*	D	PM	0.1	0/39/ 241700	SRNS	MCNS	7	F	Turkish	No	B2484	No	VUS
PLCE1	B3701_21	c.5363dup	p.Tyr1788*	Hom	*n/a*	n/a	n/a	n/a	n/p	Proteinuria	n/a	7	F	Arabic	Myopia	B3701	No	P
SMARCAL1	F1106_21	c.836T>C	p.Phe279Ser	Comp. Het	*Ci*	T	DC	0.96	0/28/282892	SRNS	FSGS	11	F	Arabic	No	F1106	Yes	VUS/ LP
SMARCAL1	F1106_21	c.1338del	p.Phe446Leufs*3	Comp. Het	*n/a*	T	DC	0.96	n/p							F1106	No	VUS/ LP
SMARCAL1	F411_21	c.2542G>T	p.[Glu848*]	Comp. Het	*n/a*	n/a	n/a	n/a	0/25/282822	NS	FSGS	13	M	Arabic	Schimke	F411	Yes	P
SMARCAL1	F411_21	c.836T>C	p.[Phe279Ser]	Comp. Het	*Ci*	n/a	n/a	n/a	0/28/282892							F411	Yes	P
SMARCAL1	F411_22	c.2542G>T	p.[Glu848*]	Comp. Het	*n/a*	n/a	n/a	n/a	0/25/282822	NS	FSGS	12	M	Arabic	Schimke	F411	Yes	P
SMARCAL1	F411_22	c.836T>C	p.[Phe279Ser]	Comp. Het	*Ci*	n/a	n/a	n/a	0/28/282892							F411	Yes	P
SMARCAL1	B1865_21	c.1940A>C	p.Lys647Thr	Hom	*Ci*	D	DC	1	n/p	SRNS	n/a	9	F	Eu/C	Recurrent UTIs	B1865	Yes	LP
TP53RK	B3326_21	c.355T>C	p.Ser119Pro	Comp. Het	*Ce*	T	DC	0.7	0/1/250834	SRNS	FSGS	7.5	M	Arabic	Facial dysmorphy	B3326	No	LP
TP53RK	B3326_21	c.128C>T	p.Ala43Val	Comp. Het	*Sc*	T	DC	0.7	n/p							B3326	Yes	LP

Supplementary table 6, cont.

Gene	Individual	Nt change c.	AA change p.	Zygosity	Cons.	SIFT	Mut. Taster	PP2	gnomAD	Clinical	Biopsy	Age (years)	Sex	Ethnicity	Syndromic	Family	Biobase	ACMG
TRPC6	**B2030_21**	**c.304T>A**	**p.Phe102Ile**	**Het**	***Ci***	**D**	**DC**	**0.99**	**0/2/276786**	**SRNS**	**FSGS**	**3**	**F**	**Eu/C**	**No**	**B2030**	**No**	**VUS/ LP**
TRPC6	**B3716_21**	**c.2294A>G**	**p.Asn765Ser**	**Het**	***Sc***	**D**	**DC**	**0.99**	**0/49/282618**	**SRNS**	**FSGS**	**14**	**F**	**Arabic**	**No**	**B3716**	**No**	**VUS**
TRPC6	**B3702_21**	**c.301C>T**	**p.Arg101Cys**	**Het**	***Ce***	**D**	**DC**	**0.9**	**0/5/244512**	**INS**	**n/a**	**0-3 m**	**F**	**Arabic**	**Facial dysmorphy**	**B3702**	**No**	**VUS**
WDR73	**B3858_21**	**c.1092dup**	**p.Ser365Leufs*25**	**Hom**	***n/a***	**n/a**	**n/a**	**n/a**	**n/p**	**SRNS**	**n/a**	**n/a**	**F**	**Arabic**		**B3858**	**No**	**P**
WT1	**A1876_21**	**c.568_586del**	**p.Pro190Alafs*95**	**Het**	***n/a***	**n/a**	**n/a**	**n/a**	**n/p**	**Wilms tumor**	**n/a**	**17**	**M**	**n/a**	**Hemi facial microsomia**	**A1876**	**No**	**VUS**
WT1	**B3843_21**	**c.1399A>G**	**p.Lys467Glu**	**Het**	***Dr***	**T**	**DC**	**P0.85**	**n/p**	**SRNS**	**FSGS**	**1**	**F**	**Arabic**	**No**	**B3843**	**No**	**VUS/ LP**
YRDC	**B2765_21**	**c.299C>G**	**p.Ser100Trp**	**Hom**	***Ce***	**D**	**DC**	**0.99**	**n/p**	**pSSNS**	**FSGS**	**7**	**F**	**Turkish**	**Microcephaly**	**B2765**	**No**	**VUS/ LP**

ACMG, American College of Medical Genetics; **AA**, amino acid; **Ce**, Caenorhabditis elegans; **Ci**, Ciona Intestinalis; **CNS**, congenital nephrotic syndrome; **Comp. Het**, compound heterozygous; **Cons.**, conservation; ; **D**, deleterious; **DC**, disease causing; **del**, deletion; **DMS**, diffuse mesangial sclerosis; **Dr**, danio rerio; **dup**, duplication; **ESRD**, end-stage renal disease; **Eu/C**, European/Caucasian; **F**, female; **fs**, frameshift; **FSGS**, focal segmental glomerulosclerosis; **Gg**, Gallus Gallus; **GGS**, global glomerulosclerosis; **gnomAD**, Genome Aggregation Database; **Het**, heterozygous; **Hom**, homozygous; **IF**, interstitial fibrosis; **INS**, infantile nephrotic syndrome; **LB**, likely benign; **LP**, likely pathogenic; ;**m**, months; **M**, male; **MCNS**, minimal change nephrotic syndrome; **Mm**, Mus Musculus; **MP**, mesangial proliferation; **Mut. Taster**, mutation taster; **n/a**, not applicable; **Nt.**, nucleotide; **P**, pathogenic; **PM**, polymorphism; **PP2**, PolyPhen-2; **pSSNS**, partial steroid sensitive nephrotic syndrome; **Sc**, Saccharomyces Cerevisiae; **SDNS**, steroid dependent nephrotic syndrome; **SIFT**, sorting intolerant from tolerant; **SRNS**, steroid resistant nephrotic syndrome; **T**, tolerated; **UTI**, urinary tract infection; **VUS**, variant of unknown significance; **Xt**, Xenopus Tropicalis.

Supplementary Table 7: Potential novel candidate genes for 57 families with steroid resistant nephrotic syndrome in whom a causative variant in a known nephrosis or phenocopy gene was excluded.

Family	Gene	Zygosity	Accesion #	c. change	p. change	conserved to	MT/SIFT/ PP2	gnom AD	Clinical Dx
B2417	*AKAP7*	Het	NM_016377.3	c.250C>T	p.Gln84*	NA	NA	NP	SRNS
B794	*ANKRD27/VARP*	hom	NM_032139.2	c.2942_2943del	p.(Leu981Glnfs*4)	NA	NA	0	SRNS
B3457	*ARFGAP3*	hom	NM_014570.4	c.1208G>A	p.(Arg403His)	Ci	DC/D/B	0	CNS
A3575	*ARSD*	hom	NM_001669.3	c.428C>T	p.(Thr143Ile)	Dm	DC/D/D	0	SRNS
B1236	*ARSD*	hom	NM_001669.3	c.428C>T	p.(Thr143Ile)	Dm	DC/D/D	0	SRNS
A1738	*BIN3*	hom	NM_018688.4	c.92T>C	p.(Leu31Pro)	Sc	DC/D/D	0	SRNS
A2283	*BSN*	Compund het	NM_003458.3	c.1555C>T; c.8486C>T	p.(Arg519Trp); p.(Thr2829Met)	Dr; Dr	D/ DC/B; D/na/na	0/ np	SRNS
A3423	*CALPN2*	hom	NM_001748.4	c.1009C>T	p.(Arg337Cys)	Dr	DC/D/D	0	SRNS
B3494	*CCDC125*	Hom	NM_176816.4	c.575G>T	p.(Arg192Ile)	Xt	DC/D/D	1	pSSNS
B1678	*CELSR1*	hom	NM_014246.1	c.3364G>A	p.(Gly1122Ser)	Dm	DC/D/D	0	SRNS
A152	*CLCA4*	hom	NM_012128.3	c.1453C>T	p.(Gln485*)	NA	NA	0	SRNS
A95	*COP1*	Compund het	NM_022457.6	c.1151G>A; c.322C>G	p.(Arg384Gln); p.(Ser108Gly)	Ci; Dr	T/DC/D; B/DC/B	np/ 0	pSSNS
A3174	*DAAM2***	Compund het	NM_015345.3	c.361G>C; c.1745C>A	p.(Glu121Gln); p.(Pro582His)	Ci; Dm	T/DC/D; B/DC/B	0/ 1	SRNS
A1635	*DACH2*	hom	NM_053281.3	c.[1397T>C]	p.[Leu466Pro]	Ci	DC/D/D	NP	SRNS
B3272	*DNM2*	hom	NM_001005360.2	c.643G>A	p.(Asp215Asn)	Dr	DC/D/B	3	pSSNS
A4401	*DOCK11*	hom	NM_144658.3	c.2571T>G	p.(His857Gln)	Dr	DC/T/D	NP	SRNS
A100	*ECPAS*	Compund het	NM_001080398.1	c.4693G>A; c.3902G>T	p.(Asp1565Asn); p.(Arg1301Leu)	Sc; np	D/DC/D; NA	np/ 0	SDNS
B1579	*EPHA3*	hom	NM_182644.2	c.621G>T	p.(Lys207Asn)	Ci	DC/D/B	0	SRNS
B2416	*EPHB2*	hom	NM_001309193.1	c.2563G>A	p.Ala855Thr	Dm	DC/D/B	0	pSSNS
B2528	*HEATR4*	hom	NM_001220484.1	c.646C>T	p.(Arg216*)	NA	NA	0	SRNS
B3626	*HSDL1*	hom	NM_173588.3	c.889A>T	p.(Ile297Phe)	Gg	DC/D/PD	NP	SRNS
A1398	*HTR1D*	Compund het	NM_000864.4	c.460C>T; c.167A>G	p.(His154Tyr); p.(Asn56Ser)	Mm; Dm	D/DC/B; D/DC/D	0/ 0	SRNS
B3750	*IGSF22*	Compund het	NM_173588.4	c.73G>A; c.296_298delCCT	p.Ser99del; p.Val25Met	Mm; NA	D/B/B; NA	0/ 0	SRNS
B3350	*ITGB8*	hom	NM_002214.2	c.1924C>A	p.(Gln642Lys)	Ci	DC/D/B	NP	SDNS
B3476	*KIRREL*	hom	NM_018240.6	c.1318C>T	p.(Arg440Cys)	Dr	DC/D/D	0	SRNS

Supplementary Table 7, cont.

A3031	*KLHL17*	hom	NM_198317.2	c.1603G>T	p.(Ala535Ser)	Xt	DC/T/D	NP	SRNS
B2486	*KLHL34*	hom	NM_153270.2	c.1006G>C	p.Val336Leu	Xt	D/T/DC	NP	SDNS
B0149	*LAMA3*	hom	NM_198129.2	c.4075T>G	p.Ser1359Ala	Xt	DC/T/D	0	CNS
B2282	*MFN2*	Compund het	NM_014874.3	c.1556G>A; c.2156A>G	p.(Arg519His); p.(Lys719Arg)	Ce; Xt	D/DC/D; T/DC/B	0/ np	pSSNS
B2287	*MPDZ*	Compund het	NM_001261407.1	c.4421A>T; c.[1301C>G]	p.[Asp1474Val]; p.[Thr434Arg]	Dr; Dr	D/DC/PD; D/DC/PD	0/ 2	SSNS
B3943	*MYO1C*	hom	NM_001080779.2	c.2273A>T	p.(Lys758Met)	Sc	DC/D/D	0	SRNS
B3314	*MYO9A***	hom	NM_006901.3	c.3910C>T	p.(Arg1304Cys)	Dr	DC/D/D	0	SRNS
A91	*P2RX4*	hom	NM_001256796.1	c.64C>T	p.(Leu22Phe)	Xt	DC/D/D	0	pSSNS
A3553	*PCDH19*	hom	NM_001184880.1	c.2098G>A	p.(Ala700Thr)	Dr	DC/D/D	0	NS
B3627	*PHKA2*	hom	NM_000292.2	c.2617C>T	p.(Leu873Phe)	Dm	DC/D/D	NP	pSSNS
B1869	*PLA2G7*	hom	NM_005084.3	c.109+2T>C	SPLICE	NA	NA	0	SRNS
B1794	*PLEC*	Compund het	NM_201380.2	c.1798C>T; c.7455G>C	p.Arg600Trp; p.Glu2485Asp	Dr; Dr	D/DC/PD; D/DC/D	0/ 0	n/a
A2908	*PLXNB3*	hom	NM_001163257.1	c.5390C>T	p.(Ser1797Leu)	Sc	DC/D/D	4	SRNS
B3541	*PLXNB3*	hom	NM_001163257.1	c.4552G>A	p.(Gly1518Ser)	Dr	DC/D/D	0	SRNS
B3855	*PLXNB3*	hom	NM_001163257.1	c.5183A>G	p.(Lys1728Arg)	Dm	D/DC/D	3	SRNS
A3595	*PPFIA1*	hom	NM_003626.3	c.3372_3374del	p.(Arg1125del)	NA	NA	0	pSSNS
B3468	*PRDM16*	hom	NM_022114.3	c.2447A>G	p.(Asn816Ser)	Dr	DC/D/D	0	SRNS
B3492	*PTH2R*	hom	NM_005048.3	c.289+1G>T	SPLICE	NA	NA	0	SRNS
A3735	*PTTG1IP*	hom	NM_004339.3	c.179G>T	p.(Cys60Phe)	Ci	DC/D/D	NP	SRNS
A3177	*RASA4B*	hom	NM_001277335.1	c.1028G>A	p.(Arg343Gln)	Sc	DC/D/D	0	SSNS
B3731	*RPS6KA6*	hom	NM_014496.5	c.1630C>G	p.(Leu544Val)	Ce	D/DC/D	0	SRNS
B1813	*SKIL*	hom	NM_005414.4	c.929C>T	p.Ser310Leu	Dr	DC/T/B	NP	Infantile NS
B2489	*SNX29*	hom	NM_032167.3	c.967T>C	p.(Ser323Pro)	Dr	D/DC/D	0	SRNS
B2604	*SORC3*	hom	NM_014978.2	c.695G>A	p.(Arg232Lys)	Dr	DC/D/D	NP	SRNS
B3137	*SYNPO2***	hom	NM_015319.2	c.3370A>T	p.(Lys1124*)	NA	NA	NP	CNS
A1942	*SYT2*	hom	NM_001136504.1	c.986A>G	p.(Lys329Arg)	Dm	DC/D/D	1	SDNS
B3482	*TBC1D8B*	hom	NM_017752.2	c.1383G>A	p.(Trp461*)	NA	NA	2	SRNS
B3328	*TLN1*	hom	NM_006289.3	c.5964_5966del	p.(Ile1989del)	NA	NA	NP	pSSNS
A3281	*XPNPEP2*	hom	NM_003399.5	c.1222_1224del	p.(Glu408del)	NA	NA	0	SRNS
B3740	*STRN*	het-de-novo	NM_003162.3	c.1859_1860del	p.(Val620Glyfs*6)	na	na	NP	SRNS
B2517	*LMLN*	het-de-novo	NM_001136049.2	c.297_298del	p.(Tyr100*)	na	na	NP	SRNS
A93	*KCNH3*	het-de-novo	NM_012284.2	c.1053del	p.(Tyr352Thrfs*9)	na	na	NP	SRNS

B, Benign; **Ce**, *Caenorhabditis elegans*; **Ci**, *Ciona intestinalis*; **CNS**, congenital nephrotic syndrome; **D**, deleterious, **DC**, disease causing; **Dr**, *Danio rerio*; Dm, *Drosophila melanogaster*; **Dx**, diagnosis; **MT**, mutation taster; **na**, not available; **NA**, not applicable; **NS**, nephrotic syndrome; **pSSNS**, partial steroid sensitive nephrotic syndrome; **PP2**, Polyphen-2; **Sc**, *Saccharomyces cerevisiae*; **SDNS**, steroid dependent nephrotic syndrome; **SIFT**, sorting intolerant from tolerant; **SSNS**, steroid sensitive nephrotic syndrome; **SRNS**, steroid resistant nephrotic syndrome; **candidate genes that were functionally characterized and recently published as potential causes of SRNS.

Supplementary Table 8: Heterozygous *de-novo* variants in novel candidate genes in 10 families with steroid resistant nephrotic syndrome in whom a likely causative variant in a known SRNS or phenocopy gene was not detected.

Family	Gene	Accesion #	c. change	p. change	conserved to	MT/SIFT/PP2	gnomAD	Clinical Dx
B3740*	*STRN*	NM_003162.3	c.1859_1860del	p.(Val620Glyfs*6)	NA	NA	NP	SRNS
B2517*	*LMLN*	NM_001136049.2	c.297_298del	p.(Tyr100*)	NA	NA	NP	SRNS
A93*	*KCNH3*	NM_012284.2	c.1053del	p.(Tyr352Thrfs*9)	NA	NA	NP	SRNS
B3866	*LRP1*	NM_002332.1	c.2798-1G>C	//	NA	NA	NP	SDNS
B2506	*RELN*	NM_005045.4	c.5797G>A	p.(Gly1933Ser)	*Xt*	D/DC/D	NP	CNS
B2413	*SYTL5*	NM_001163334.1	c.1415A>G	p.(Tyr472Cys)	*Dr*	D/DC/D	NP	SDNS
B3398	*POLR2A*	NM_000937.4	c.5692A>G	p.(Thr1898Ala)	*Ce*	D/DC/PD	NP	SDNS
B3859	*ANKIB1*	NM_019004.1	c.2303A>G	p.(Tyr768Cys)	*Ce*	D/DC/D	NP	SRNS
B4139	*PHF6*	NM_001015877.1	c.969-2A>C	p.? (splice site)	NA	NA	NP	NRP
B3922	*SIGLEC1*	NM_023068.4	c.4844C>A	p.(Ala1615Asp)	*Xt*	B/DC/D	NP	SRNS

Ce, *Caenorhabditis elegans;* **CNS**, congenital nephrotic syndrome; **D**, deleterious; **DC**, disease causing; **Dr**, *Danio rerio*; **Dx**, diagnosis; **Het**., heterozygous; **MT**, mutation taster; **NA**, not applicable; **PD**, probably deleterious; **PP2**, Polyphen-2; **SDNS**, steroid dependent nephrotic syndrome; **SIFT**, sorting intolerant from tolerant; **SRNS**, steroid resistant nephrotic syndrome; **Xt**, *Xenopus tropicalis*.
* In these families, no other likely causative variant was found, and they were therefore defined as ‘novel candidate genes’. In the other seven families in which de-novo variants were found, one or more additional pathogenic (inherited) variants were found, so their ES analysis was defined as ‘inconclusive’.

Supplementary Table 9: Presence of homozygosity, analysis structure and extra-renal manifestations in 320 families with steroid-resistant nephrotic syndrome.

A subset of 88 families, in whom a likely causative variant was detected in a SRNS gene or of 18 families in whom a causative variant was detected in a phenocopy gene are shown. Also shown, is pedigree structure based on DNA availability, and the presence of extra-renal manifestations in 343 individuals from 320 families with SRNS.

	Clinical charac-teristics of total cohort	Clinical characteristics of individuals in whom likely causative variant detected	
	Number of families (%)	Number of families with likely causative SRNS variant detected (%)	Number of families with likely causative variant detected in phenocopy gene (%)
**Pedigree**			
**Homozygosity ≥50Mb**	**101 (31.6%)**	**52 (59%)**	**4 (22.2%)**
**Homozygosity <50Mb**	**219 (68.4%)**	**36 (41%)**	**14 (77.8%)**
**Total**	**320 (100%)**	**88 (100%)**	**18 (100%)**
**Pedigree structure**	**Number of families (%)**	**Number of families with likely causative SRNS variant detected (%)**	**Number of families with likely causative variant detected in phenocopy gene (%)**
**Singlet**	**196 (61.2%)**	**51 (57.9%)**	**11 (61.1%)**
**Multiple affected**	**13 (4.0%)**	**5 (5.6%)**	**1 (9.0%)**
**Duo**	**30 (9.3%)**	**5 (5.6%)**	**2 (18.1%)**
**Trio**	**81 (25.3%)**	**27 (30.6%)**	**4 (36.3%)**
**Total**	**320 (100%)**	**88 (100%)**	**18 (100%)**
**Extra-renal manifestations**	**Number of individuals (%)**	**Number of individuals with likely causative SRNS variant detected (%)**	**Number of individuals with likely causative variant detected in phenocopy gene (%)**
**Yes**	**77 (22.4%)**	**31 (31%)**	**6 (28.6%)**
**No, or not reported**	**266 (77.6%)**	**69 (69%)**	**15 (71.4%)**
**Total**	**343 (100%)**	**100 (100%)**	**21 (100%)**

Mb, mega-bases; SRNS, steroid-resistant nephrotic syndrome.

Supplementary Table 10: Clinical and histologic diagnosis of 343 individuals from 320 families with steroid-resistant nephrotic syndrome. A subset of 100 individuals from 88 families, in whom a causative variant was detected in a steroid-resistant nephrotic syndrome gene and 21 individuals from 18 families, in whom a phenocopy gene was detected are shown.

	Clinical characteristics of total cohort	Clinical characteristics of individuals with causative variant detected	
	Number of individuals (%)	Number of individuals with SRNS variant detected (%)	Number of individuals with variant detected in phenocopy gene (%)
**Inclusion by Clinical Diagnosis or biopsy findings**			
**SRNS**	**210 (61.2%)**	**50 (50%)**	**13 (61.9%)**
**CNS (no steroids given)**	**36 (10.5%)**	**24 (24%)**	**0 (0%)**
**Infantile (no steroids given)**	**16 (4.7%)**	**13 (13%)**	**1 (4.8%)**
**SDNS**	**23 (6.7%)**	**1 (1%)**	**2 (9.5%)**
**pSSNS**	**31 (9%)**	**2 (2%)**	**1 (4.8%)**
**FSGS/DMS**	**19 (5.5%)**	**6 (6%)**	**2 (9.5%)**
**Included based on family member inclusion**	**8 (2.3%)**	**4 (4%)**	**2 (9.5%)**
**Total**	**343 (100%)**	**100 (100%)**	**21 (100%)**
**Diagnosis on renal Biopsy**			
**FSGS**	**106 (30.9%)**	**30 (30%)**	**5 (23.8%)**
**DMS**	**8 (2.3%)**	**5 (5%)**	**1 (4.8%)**
**MPGN**	**26 (7.6%)**	**2 (2%)**	**6 (28.6%)**
**MCNS**	**40 (11.7%)**	**5 (5%)**	**0 (0%)**
**Other biopsy**	**18 (5.2%)**	**1 (1%)**	**2 (9.5%)**
**Biopsy N/A**	**145 (42.5%)**	**57 (57%)**	**7 (33.3%)**
**Total**	**343 (100%)**	**100 (100%)**	**21 (100%)**

CNS, congenital nephrotic syndrome; **DMS**, diffuse mesangial sclerosis; **FSGS**, focal segmental glomerulosclerosis; **MCNS**, minimal change nephrotic syndrome; **MPGN**, membranoproliferative glomerulonephritis; **pSSNS**, partial steroid-sensitive nephrotic syndrome; **SDNS**, steroid-dependent nephrotic syndrome; **SRNS**, steroid resistant nephrotic syndrome.

Supplementary Table 11: Summary of whole exome sequencing results in patients with SRNS across various research groups and cohorts.

									Rate of detection of likely causative variants in a known SRNS gene								
**First author (reference # in bibliography)**	**number of families**	**Number of individuals with SRNS included in analysis**	**Number of known SRNS genes screened**	**Age of onset of SRNS (inclusion criterion, median), years**	**Median age (of individuals for whom a causative variant was detected), years**	**Median age (of individuals for whom a causative variant was not detected), years**	**Percent of consanguinous families %**	**Percent of families with congenital or infantile SRNS %**	**Overall (%)**	**In consanguinous families %**	**In non-consanguinous families %**	**In congenital or infantile NS %**	**Most frequent known SRNS genes in which likely causative variants were found**	**Detection rate of a likely causative variant in a phenocopy gene %**	**Detection rate of a causative variant in single potential novel candidate gene %**	**Inconclusive (detection of multiple candidates) %**	**Unsolved %**
**Current work**	**320**	**343**	**59**	**<25, 3**	**2**	**4**	**31.6**	**21.5**	**27.5**	**50.9**	**18.0**	**65.5**	***NPHS1, LAMB2, NPHS2, PLCE1, COQ8/ADCK4***	**5.6**	**18.8**	**12.5**	**35.0**
**Warejko (3)**	**300**	**335**	**33**	**<25, 3**	**1.7**	**4**	**49**	**31**	**25.0**	**38**	**13**	**38**	***NPHS1, PLCE1, NPHS2, SMARCAL1, LAMB2***	**3.7**	**28 ***		**44.0**
**Sadowski (1)**	**1,783**	**2,016**	**27**	**<25, 3.5**	**n/a**	**n/a**	**20.9**	**22.3**	**29.5**	**49.5**	**25**	**61.3**	***NPHS1, LAMB2, PLCE1, NPHS2, WT1***	**n/a**	**n/a**	**n/a**	**70.5**
**Landini (38)**	**111**	**64**	**298 ****	**<30, 4**	**n/a**	**n/a**	**n/a**	**n/a**	**30**	**n/a**	**n/a**	**n/a**	***NPHS2, NPHS1, ANLN, PLCE1, ACTN4***	**28**	**n/a**	**n/a**	**26**
**Sen (39)**	**302**	**255 (46 adults)**	**27**	**not restricted,**	**n/a**	**n/a**	**12.1**	**24.5**	**21.2**	**38.5**	**26.9**	**58.1**	***NPHS1, WT1, NPHS2, LMX1B, LAMB2***	**n/a**	**n/a**	**n/a**	**78.8**
**Byerzynska (40)**	**187**	**187**	**53**	**>19**	**n/a**	**n/a**	**7**	**11.2**	**26.2§**	**n/a**	**n/a**	**76**	***NPHS1, NPHS2, WT1, TRPC6, LAMB2***	**n/a**	**n/a**	**n/a**	**73.8**

* Potential 'single novel' and 'multiple candidate' genes categories were combined; ** Kidney-disease genes; § Including phenocopy genes

Supplementary table 12: Genes discovered in Warejko CJASN 13:53, 2018 to be potential candidate SRNS genes, and which were subsequently studied for functional deleteriousness and published.

Gene	Reference
TPRKB	Braun Nat Genet 49:1529, 2017
OSGEP	Braun Nat Genet 49:1529, 2017
AVIL	Rao J Clin Invest 1:4257, 2017
ITSN1	Ashraf Nat Commun 17:1960, 2018
TENC1	Ashraf Nat Commun 17:1960, 2018
CDK20	Ashraf Nat Commun 17:1960, 2018
GAPVD1	Hermle J Am Soc Nephrol 29:2123, 2018
LAMA5	Braun Nephrol Dial Transplant 1:485, 2019
KIRREL1	Solanki Kidney Int 96:883, 2019
TBC1D8B	Kampf J Am Soc Nephrol 30:2338, 2019
DAAM2	Schneider Am J Hum Genet 3:1113, 2020
NOS1AP	Majmundar Sci Adv 1;7, 2021
PRDM15	Mann J Am Soc Nephrol32:580, 2021
