# Supplementary material for: Expanding the spectrum of novel candidate genes using trio exome sequencing and identification of monogenic cause in 27.5% of 320 families with steroid-resistant nephrotic syndrome
Source: Genes Dis. 2024 Mar 28;12(2):101280. doi: 10.1016/j.gendis.2024.101280 (PMC11582537; doi:10.1016/j.gendis.2024.101280)
Supplement: Multimedia component 3 [file mmc3.docx]

**Supplementary data:**

**MATERIALS AND METHODS**

**Human participants.** The study was approved by the institutional review board (IRB) of the University of Michigan and Boston Children’s Hospital. From April 1998 to December 2018, individuals were enrolled after obtaining informed consent. Inclusion criteria were start of a clinical diagnosis of SRNS and any of the 3 findings before the age of 25 years: i) steroid-dependent nephrotic syndrome (SDNS), ii) nephrotic range proteinuria with renal histology of focal segmental glomerulosclerosis (FSGS), or iii) diffuse mesangial sclerosis (DMS) (**sTable 1**). We also included individuals with disease onset before age 3 months (congenital nephrotic syndrome) or onset before 1 year of age, since these patients are considered conceptually steroid-resistant even if no treatment attempt has been made. They are therefore typically not treated with steroids. A total of 343 individuals from different 320 families (affected siblings were in the analysis) were enrolled and examined for exome sequencing (ES). Before December 2013, enrolled individuals had been screened for variants in *WT1* and *NPHS2.* The individuals whom we found positive were not included in this study. Individuals included in our previous ES studies were excluded from this study.

Our population was ethnically diverse (**2**). The most frequently represented groups were of Arab, Asian, Caucasian, or Turkish descent. Individuals from Saudi Arabia and those from Egypt were identified as “Arabic” (**sTable 2)**.

**Exome sequencing and variant calling.** Exome sequencing (ES) and variant burden analysis were performed as previously described^1-3^. In brief, genomic DNA was isolated from blood lymphocyte or saliva samples and subjected to exome capture using Agilent SureSelect™ human exome capture arrays (Life technologies™) followed by next- generation sequencing on the Illumina HighSeq™ sequencing platform. Sequence reads were mapped to the human reference genome assembly (NCBI build 37/hg19) using CLC Genomics Workbench™ (version 6.5.2) software (CLC bio, Aarhus, Denmark). Following alignment to the human reference genome (GRCh37/hg19), variants were filtered for most likely non-deleterious variants as previously described [[7](#_ENREF_7), [10](#_ENREF_10)]. In the first step, variants with minor allele frequencies >1% in the dbSNP (version 142) or in the 1,000 Genomes Project (1,094 subjects of various ethnicities; May 2011 data release) databases were excluded as they were unlikely to be deleterious. We used manual inspection for the p.Arg229Gln variant in the *NPHS2* gene which is reported to be deleterious with other variants, which has a high frequency in the population and would be filtered out using this method. Synonymous variants and intronic variants that were not located within splice site regions were excluded. The remaining variants included non-synonymous variants and splice site variants.

**Variant calling in known SRNS genes.** We evaluated ES data for causative variants in 59 monogenic SRNS-causing genes (**sTable 3**). Variant calling was applied as stated above, followed by filtering of remaining variants for changes in the regions of the 59 SRNS genes (**sTable 4)**. The remaining variants were ranked based on their probable impact on the function of the encoded protein considering evolutionary conservation among orthologues across phylogeny using ENSEMBL Genome Browser and assembled using Clustal Omega, as well as web-based prediction programs PolyPhen-2, SIFT, and Mutation Taster [[16](#_ENREF_16)]. We excluded variants if their biallelic frequency in the Genome Aggregation Database (gnomAD) was ~5 or higher. Variant calling was performed by clinician-scientists/geneticists in our laboratory, who had knowledge of the clinical phenotypes and pedigree structure, as well as experience with ES evaluation. Remaining variants were confirmed in patient DNA by Sanger sequencing as previously described [[7](#_ENREF_7)]. Finally, pathogenicity value was assigned to each variant using ACMG criteria (sTable 6) [[17](#_ENREF_17)]. Whenever parental DNA was available, a duo or trio analysis using the CLC platform was performed.

If no causative variant was identified, we also evaluated for variants in 15 genes that may represent phenocopies of SRNS (*COL4A3, COL4A4, COL4A5, DGKE, FN1,MEFV, NOTCH3, THBD, ADAMTS13, C3, CD46, CDK20, CFH, CFHR5,* and *CFI*) (**sTable 5**). Variants were evaluated as above. A correlation of genotype and phenotype was examined and, if congruent, the genetic variant was deemed likely causative.

**Variant calling to identify novel causes of SRNS.** If no likely causative variant was found in a known SRNS gene. Single heterozygous variants were only analyzed in trios for potential *de-novo* variation. Remaining variants were ranked based as described above, using conservation across phylogeny and web-based prediction programs as to their impact on protein function. In families with a low degree of homozygosity detected by mapping (<50 Mbp) we have evaluated all homozygous calls (regardless of homozygous peaks). When parental DNA was available, we evaluated all compound heterozygous variants as well as potential de-novo variants. Variants were confirmed in original patient and parental DNA by Sanger sequencing as previously described [[7](#_ENREF_7)].

**COHORT CHARACTERISTICS**

For 207 families, we had only the affected child’s DNA available for analysis (singlets), for 32 families we had the DNA of one of the parents as well (duos), and for 81 families, we had both parental DNA for analysis (trios). 13 of the families had more than one affected individual.

The onset of SRNS in our cohort ranged from birth to 20 years of age. 56.7% of cases had onset of SRNS before 6 years of age (**sTable 2**). The age range in families in whom we detected a causative variant was 0-17 years (**sFig 1**). 63.3% of probands with causative variants detected in an SRNS gene were under 6 years of age. The median age in those with causative variants detected in an SRNS gene was 2 years versus 4 years in those without a causative variant identified (**sTable 2**).

We evaluated each family’s ES data for homozygosity. Affected individuals from 101 families were found to have ≥50 Mbp of homozygosity on mapping (31.6%) and affected individuals from 219 families were found to have homozygosity of <50 Mb by mapping.

We reviewed each individual for extra-renal manifestations, such as cardiac defects, microcephaly, and facial dysmorphism. 77 of 343 (22.4%) patients had extra-renal manifestations (**sTable 9**). 31% of individuals for whom a causative variant in a known SRNS gene was found, had extra-renal manifestations. (**sTable 9**), and 69% of individuals with no molecular diagnosis in an SRNS gene, did not have extra-renal manifestations, or these were not reported. In 40.2% with and 25.9% of those with no extra-renal manifestations, a causative variant was detected in an SRNS gene (p=0.02, Fisher Exact test).

In our cohort, the most common clinical diagnosis was SRNS in 210/343 (61.2%) compared to those who were diagnosed with congenital nephrotic syndrome, infantile nephrotic syndrome, steroid-dependent, or partial steroid-sensitive NS (**sTable 10**). SRNS was also the most common clinical diagnosis in those families with a causative variant identified (50 of 88 families, 56.8%), (**sTable 10**).

In 198/343 (57.7%) individuals of 188 families, renal biopsy data were available. 30% of all solved cases for known SRNS genes, had FSGS on kidney biopsy (**sTable 10**). Out of 8 individuals with DMS on biopsy, a likely deleterious variant in an SRNS gene was identified in 5.

Web Resources

WEB RESOURCES

1000 Genomes Browser, http://browser.1000genomes.org

ClinVar, http://www.ncbi.nlm.nih.gov/clinvar

Clustal Omega, http://www.ebi.ac.uk/Tools/msa/clustal

dbSNP, http://www.ncbi.nlm.nih.gov/snp

Ensembl Genome Browser, http://www.ensembl.org

Exome Variant Server, http://evs.gs.washington.edu/EVS

Genome Aggregation Database (gnomAD), http://gnomad.broadinstitute.org

HGMD® Professional 2020.4, http://portal.biobase-international.com/hgmd

Mouse Genome Informatics, http://www.informatics.jax.org

MutationTaster, http://www.mutationtaster.org

Online Mendelian Inheritance in Man (OMIM®), http://www.omim.org

PolyPhen2, http://genetics.bwh.harvard.edu/pph2

Sorting Intolerant From Tolerant (SIFT), http://sift.jcvi.org

UCSC Genome Browser, http://genome.ucsc.edu/cgi-bin/hgGateway

Uniprot Consortium, http://www.uniprot.org

VarSome, http://www.varsome.com
